# Zinc Silicate-Loaded Microneedle Patch Reduces Reactive Oxygen Species Production and Enhances Collagen Synthesis for Ultraviolet B-Induced Skin Repair

**DOI:** 10.34133/bmr.0180

**Published:** 2025-04-10

**Authors:** Fang-Zhou Chen, Zhao-Wen-Bin Zhang, Qing-Feng Li, Poh-Ching Tan, Jiang Chang, Shuang-Bai Zhou

**Affiliations:** ^1^ Department of Plastic and Reconstructive Surgery, Shanghai Jiao Tong University School of Medicine Affiliated Ninth People’s Hospital, Shanghai, China.; ^2^ Joint Centre of Translational Medicine, The First Affiliated Hospital of Wenzhou Medical University, Wenzhou 325000, China.; ^3^ Zhejiang Engineering Research Center for Tissue Repair Materials, Wenzhou Institute, University of Chinese Academy of Sciences, Wenzhou 325000, China.; ^4^ State Key Laboratory of High-Performance Ceramics and Superfine Microstructure, ShanghaiInstitute of Ceramics, Chinese Academy of Sciences, Shanghai 200050, China.; ^5^ College of Biological Science and Medical Engineering, Donghua University, Shanghai 201620, China.

## Abstract

UVR-related skin damage is common in daily life. Excessive sunlight exposure, particularly in response to ultraviolet B (UVB) radiation, can have adverse effects on the skin and can even induce photosensitive skin diseases and skin malignancies. UVB exposure leads to the production of reactive oxygen species (ROS) in the skin, resulting in cell damage and inflammation. Furthermore, it directly inhibits the synthesis of collagen in skin fibroblasts, contributing to collagen degradation and subsequently causing skin aging, wrinkles, and erythema. To address this issue, our study introduces a biomaterial-based treatment plan for repairing UVB-induced photodamaged skin. We designed a sodium hyaluronate microneedle patch containing a hardystonite bioceramic (ZnCS/MN) with anti-ROS/inflammation/collagen degradation functions to deliver bioactive Zn^2+^ and SiO_3_^2−^ ions in situ to photodamaged skin areas. In addition, the cytological mechanism of ZnCS action was explored to explore the possibilities of its application in more areas. This study reveals the therapeutic potential of ZnCS for a variety of negative effects caused by photodamage. Owing to its advantages in preparation, storage, and transportation, ZnCS/MN has shown promise for clinical application in treating photodamaging.

## Introduction

The skin, as the largest organ of the body, plays a critical role in acting as a protective barrier against the external environment [1,2]. However, prolonged exposure to ultraviolet radiation (UVR) results in skin photodamage [3]. This process of damage is characterized by a series of pathological changes, including the development of wrinkles, erythema, pigmentation, and increased susceptibility to malignant skin tumors [4,5]. The application of retinoids combined with dermal filler surgery is the most common treatment for skin photodamage in the clinic [6–8]. However, it is crucial to recognize that these treatments have several limitations. The use of retinoids, for example, can result in skin irritation when exposed to UV rays, potentially increasing skin damage [6,7,9]. Furthermore, surgical intervention is associated with adverse effects, including skin discomfort, darkening, and erythema [8]. Additionally, these treatment modalities primarily focus on symptom relief rather than addressing the underlying mechanisms of photodamage. To solve this problem, researchers have been developing novel strategies, such as stem cell therapy, for the treatment of skin photodamage [10,11]. However, these patients still face safety and ethical issues [12]. Thus, there is a need for the development of secure, convenient, and effective approaches to address skin photodamage.

Abnormal accumulation of reactive oxygen species (ROS) and the resulting histological damage (collagen degradation) are the key factors in skin photodamage. These ROS are induced by UVR, particularly UVB radiation (280 to 315 nm) [13], which activates multiple signaling pathways, including the senescence-associated secretory phenotype (SASP). As a consequence, skin fibroblasts lose vitality [14] and exhibit reduced or halted collagen secretion, which leads to deteriorated skin texture and aging of the skin [15]. Therefore, eliminating excess ROS and promoting collagen secretion in skin fibroblasts are both key concerns in the treatment of skin photodamage. The identification of treatments that have multiple effects on the inhibition of ROS production and the promotion of collagen content could lead to better results.

In recent years, silicate bioceramics have emerged as promising biomaterials for tissue repair due to their ability to modulate the physiological microenvironment through the controlled release of bioactive ions [16,17]. Silicate ions (SiO_3_^2−^) have been shown to markedly increase the viability of various cell types, including fibroblasts, cardiomyocytes, and vascular endothelial cells [18–20]. This property makes it effective at treating many types of tissue injuries, ranging from diabetic wounds to myocardial infarction [21,22]. However, while silicate bioceramics have demonstrated potential in promoting skin regeneration, their clinical application remains limited by insufficient control over ion release and the need for improved biocompatibility in transdermal applications. Zinc ions (Zn^2+^) play crucial roles in the antioxidant defense system, contributing to the maintenance of an anti-inflammatory microenvironment during various tissue repair processes, including skin repair [23,24]. Additionally, Zn^2+^ actively participates in mitochondrial reduction reactions and effectively eliminates excess ROS [25]. By combining the regenerative properties of silicate bioceramics with the antioxidant and anti-inflammatory functions of Zn^2+^, a dual-functional biomaterial could be developed to address key challenges in photodamaged skin repair.

**Fig. 1.**
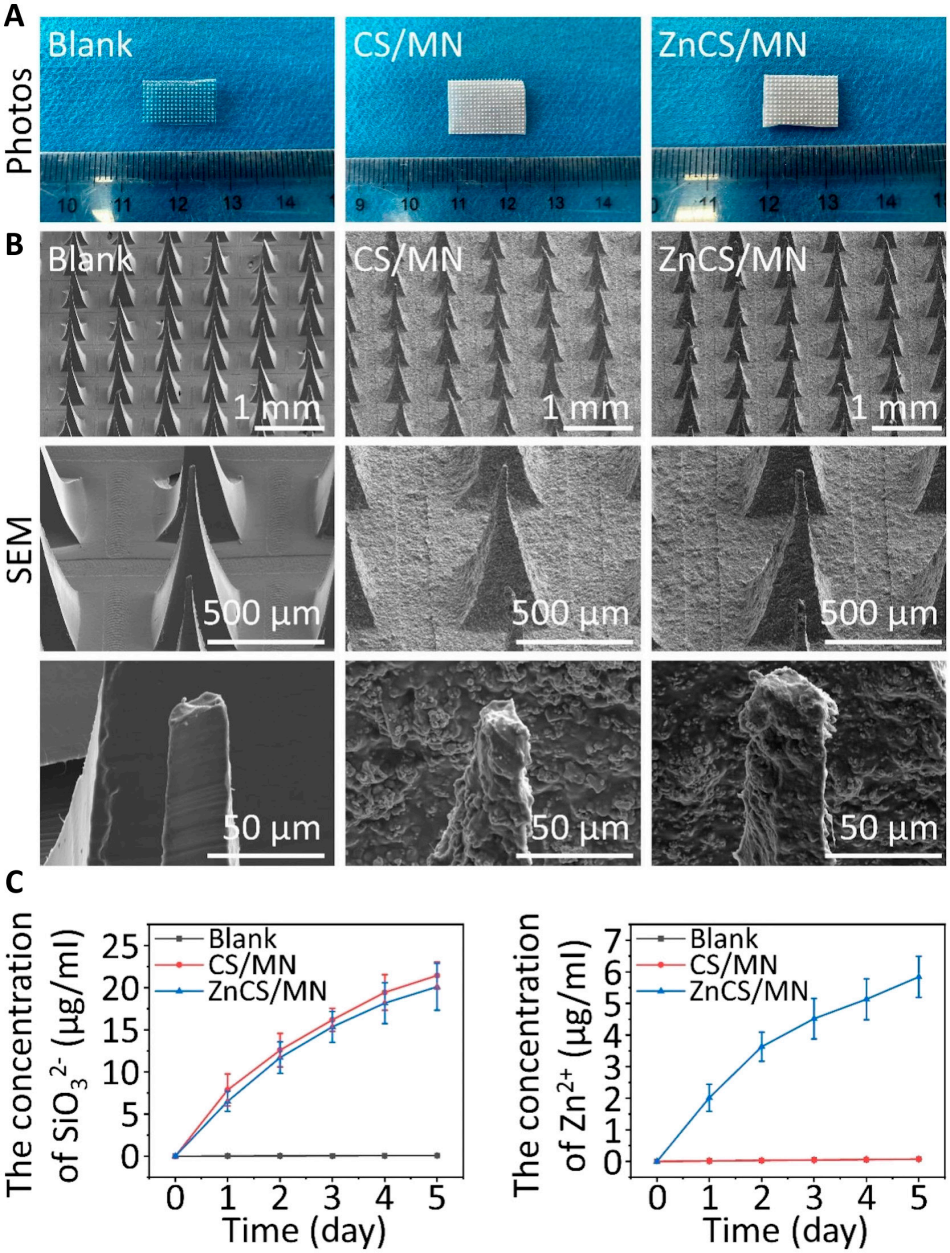
Characterization of microneedles. (A) Optical photographs of microneedles. (B) SEM images of the microneedle array, single microneedle, and microneedle tip. Scale bars, 1,000 μm (array), 500 μm (single microneedle), and 50 μm (tip). (C) SiO_3_^2−^ and Zn^2+^ ion release profile of the microneedles after soaking in PBS at 37 °C for 5 d. Statistical analysis was performed using one-way ANOVA followed by Tukey’s HSD test (*P < 0.05, **P < 0.01, ***P < 0.001). Blank: sodium hyaluronate microneedles; CS/MN: sodium hyaluronate microneedles containing CS bioceramics; ZnCS/MN: sodium hyaluronate microneedles containing ZnCS bioceramics.

In this study, we developed a sodium hyaluronate composite microneedle patch (ZnCS/MN) containing Zn-silicate bioceramics (hardystonite, ZnCS) for delivering SiO_3_^2−^ and Zn^2+^ ions released from hardystonite into the dermal layer. By integrating silicate bioceramics into a microneedle-based delivery system, ZnCS/MN overcomes the limitations of conventional bioceramic applications by ensuring controlled ion release and enhanced bioavailability. The effects of the ZnCS/MN composite MNs on photodamage were evaluated via in vivo UV photodamage in an animal model, and the related mechanisms were investigated via in vitro photodamaged cell culture experiments (Fig. 1).

## Materials and Methods

### Preparation of CS and ZnCS bioceramics

The synthesis of CS ceramic powder involves direct precipitation via the use of calcium nitrate tetrahydrate and sodium silicate nonahydrate as raw materials. Initially, a 0.5 M solution was prepared by dissolving calcium nitrate tetrahydrate and sodium silicate nonahydrate, and the pH of the calcium nitrate solution was adjusted to 11.5. The sodium silicate solution was then slowly added dropwise into the vigorously stirred calcium nitrate solution, resulting in the formation of a white precipitate. The mixture was stirred for 24 h, followed by centrifugation at 5,000 rpm, after which the supernatant was discarded, and a white precipitate was obtained. This precipitate was placed in a vacuum drying box, calcined at 800 °C for 2 h after complete drying, and then ground to obtain the requisite CS bioceramic powder.

For the preparation of hardystonite, TEOS (tetraethyl orthosilicate), H_2_O, and a 2 M HNO_3_ solution were mixed at a molar ratio of 1:8:0.16 and stirred for 30 min. Subsequently, zinc nitrate and calcium nitrate were added to the solution and stirred for 5 h, maintaining a Si:Zn:Ca ratio of 2:1:2. The mixed solution was aged in an oven at 60 °C for 24 h, followed by drying at 120 °C for 48 h. The resulting xerogel was ball milled, passed through a 200-mesh screen, and calcined at 1,200 °C for 3 h. Upon completion of sintering, the material was ball milled again and passed through a 400-mesh sieve to obtain hardystonite.

### Preparation of bioceramic composite microneedles

The microneedles were fabricated via a polydimethylsiloxane (PDMS) mold with a microneedle array dimension of 20 × 20. Briefly, 2 g of sodium hyaluronate was dissolved in 50 ml of deionized water at 37 °C. Subsequently, 0.5 g of HSA (hardystonite) bioceramic was added to the solution, followed by continuous stirring for 24 h. Afterward, 1 ml of the solution was dispensed onto the PDMS mold and subjected to vacuum drying at 37 °C for 2 h. Finally, the mold underwent demolding in an oven at 37 °C for 72 h to yield ZnCS-loaded MNs (ZnCS/MN). Pure sodium hyaluronate microneedles (Blank) and CS-loaded microneedles (CS/MN) were also prepared as control groups via the same protocol with equal amounts of the corresponding ingredients. The ion (Zn^2+^ and SiO_3_^2−^) release concentrations were measured via inductively coupled plasma–atomic emission spectrometry (ICP–AES; Thermo Fisher X Series 2, USA) from day 1 to day 5.

### Characterization of bioceramics and microneedles

The surface morphology of the bioceramics was investigated by scanning electron microscopy (SEM; S-4800, Hitachi, Japan). Additionally, the elemental contents (O, Si, Ca, and Zn) of the bioceramics were analyzed via an SEM accessory energy-dispersive spectrometer (EDS) system. Optical images of the various MNs were acquired with a camera, and their surface morphologies were further evaluated via SEM.

### Cell culture and UVB irradiation

NIH-3T3 cells (2 × 10^5^/well) were cultured in a cell culture plate (6-well) for 24 h at 37 °C in a CO_2_ incubator. After being washed with phosphate-buffered saline (PBS), the NIH-3T3 cells were incubated with 1 ml of PBS and irradiated with UVB (125 mJ/cm^2^) via a UVB lamp (Philip, 311 nm, 20 W/01, Germany). After the culture medium was replaced with serum-free Dulbecco’s modified Eagle’s medium, Zn^2+^/SiO_3_^2−^ was added, and the cells were further incubated for 24 to 72 h.

### CCK-8 assay

Cell viability was estimated by the CCK-8 (Cell Counting Kit-8) assay. Approximately 10^4^ cells were seeded in each well of 96-well plates with 100 μl of medium per well. The wells were incubated with 10 μg of CCK-8 solution for 1 h in the dark before the absorbance at 450 nm was measured with a multilabel plate reader.

### cAMP assay

3T3 cells were collected from the culture dish, and the cells were lysed by repeated freeze–thawing. The cyclic adenosine monophosphate (cAMP) antagonist SQ22536 (#HY-100396; MCE, China) was dissolved in dimethylsulfoxide (DMSO) to prepare a 1 mM working solution. SQ22536 at a final concentration of 10 μM was added to the cell culture medium. The cAMP concentrations of the collected cell lysates were measured with a cAMP enzyme-linked immunosorbent assay (ELISA) kit (Abcam, ab65355) according to the manufacturer’s protocol. The absorbance was measured at 450 nm via a microplate reader.

### Cell cycle analysis

After UVB irradiation and treatment, the NIH-3T3 cells were fixed with 75% ethanol for more than 20 h. Then, the cells were washed with PBS and stained with propidium iodide (PI). The cell cycle distribution was analyzed via flow cytometry (BD).

### Detection of the intracellular mitochondrial membrane potential 

The cells were subjected to staining with 2 μM JC-1 at 37 °C in a dark environment for 20 min. This was accomplished via the use of a Mitochondrial Membrane Potential Assay Kit with JC-1 (Beyotime Biotechnology). The cells were subsequently visualized and imaged via a microscope equipped with a digital camera.

### Animal study

All procedures were approved by the Animal Research Committee of Shanghai Jiao Tong University Affiliated Ninth People’s Hospital (SH9H-2023-A879-SB) and conducted in compliance with institutional and national ethical guidelines for animal care. A total of 32 female BALB/c nude mice (5 weeks old) were raised in a specific pathogen-free (SPF) environment under controlled conditions for 1 week to adapt to the new environment . Mice were housed in individual ventilated cages (IVCs) at a temperature of 22 ± 2 °C, 50 ± 10% relative humidity, and a 12-h light/dark cycle, with ad libitum access to sterilized food and water. After the adaptation period, they were randomly assigned to 4 groups (*n* = 8): (a) the control group: no UVB irradiation or treatment; (b) the UVB + Blank group: UVB irradiation + no-load MN patches; (c) the UVB + CS/week group: UVB irradiation + CS-containing MN patches; (d) the UVB + ZnCS group: UVB irradiation + ZnCS-containing MN patches.

Nude mice were irradiated with a UVB lamp (Philip, 311 nm, 20 W/01, Germany) for 8 weeks [48]. The irradiation dose was one minimal erythema dose (MED) of 160 mJ/cm^2^ in the first week, followed by 210, 280, and 370 mJ/cm^2^ in weeks 2 to 4 and 370 mJ/cm^2^ in weeks 5 to 8. The energy density was measured with a UVB energy detector (UV-DETECTOR 150, Ergu, China). Interventions were held during the fourth and sixth weeks. Microneedle (MN) patches were applied immediately after UVB irradiation in the corresponding treatment groups to ensure optimal absorption and therapeutic effect.

### Histological analysis of skin tissue

The isolated dorsal skin tissues were fixed in 10% paraformaldehyde solution and embedded in paraffin. The skin tissue was sliced (5 μm) and visualized via hematoxylin and eosin (H&E) staining. Masson’s trichrome staining was used to determine the density of the collagen fibers. A high-magnification microscope and an image analysis system (DS-Ri2, Nikon, Tokyo, Japan) were used for visualization and analysis. Skin thickness and collagen density were analyzed via the ImageJ program.

### Sirius red staining

Sirius red staining was performed, and the type of collagen present was determined under polarized light (DS-Ri2, Nikon, Tokyo, Japan): intense yellow–red birefringence indicates type I collagen, and weak green birefringence indicates type III collagen [49]. The type III collagen/type I collagen ratio, collagen content, and proportion of mature collagen were quantified via color deconvolution via ImageJ v1.53k with the Color Deconvolution plugin (H-DAB mode). Regions of interest (ROIs) were manually selected across the dermal layer, and thresholding (0 to 150 for green, 151 to 255 for red) was applied to isolate collagen subtypes. Collagen content was calculated as the percentage of positively stained area relative to total dermal area, while the type III/I ratio was derived from their respective pixel densities.

### Senescence β-galactosidase staining

The rewarmed frozen tissue sections were washed 3 times with PBS, stained via a Senescence β-Galactosidase Staining Kit (Beyotime, C0602), incubated with an appropriate volume of β-galactosidase staining fixative, and washed again 3 times with PBS, after which the appropriate amount of staining working solution was added. The preparation method was performed according to the manufacturer’s instructions. The samples were incubated at 37 °C overnight. Images were observed under a light microscope, and the number of positive cells was counted via ImageJ.

### Immunohistochemical staining

Individual dorsal skin samples were collected on the first day after modeling (8 weeks) when the experiments were terminated. After being rinsed 3 times with PBS and incubated in 0.3% Triton X-100 and 3% bovine serum albumin for 30 min at 37 °C, the sections were incubated with the primary antibody. Rabbit anti-γH2AX (1:300; 9718; Cell Signaling) was incubated at 4 °C overnight, followed by incubation with the secondary antibody donkey anti-rabbit AF488 (1:1,000; A21206; Thermo Fisher). Then, the cell nuclei were stained with 4′,6-diamidino-2-phenylindole (DAPI) (Beyotime, China). Images were acquired via a laser confocal scanning microscope and analyzed for fluorescence intensity via ImageJ.

### Measurement of ROS production

At the end of UVB irradiation, the NIH-3T3 cells were washed with PBS, DHE (dihydroethidium, a red fluorescent reactive oxygen probe) was added, and the cells were further incubated for 30 min at 37 °C in a CO_2_ incubator. The cells were then washed with precooled PBS, and DHE positivity was measured via flow cytometry.

The frozen tissue sections were incubated with DHE for 30 min at 37 °C in a CO_2_ incubator. The sections were washed 2 to 3 times with PBS and sealed, and the images were observed via a fluorescence microscope at an excitation wavelength of 535 nm.

### Reverse transcription and real-time quantitative reverse transcription polymerase chain reaction

Total RNA was extracted from mouse skin tissue and NIH-3T3 cells via TRIzol reagent. One microgram of total RNA and 10 pM oligo dT primer were used to synthesize complementary DNA (cDNA). Gene expression was evaluated via quantitative polymerase chain reaction (qPCR) performed on a real-time 7500 HT system (Applied Biosystems, Foster City, CA) via the SYBR Green PCR Master Mix Kit (Takara Bio). The annealing temperature for PCR was set at 60 °C. The relative expression of each gene was evaluated via the *C*_t_ method and normalized to the expression of glyceraldehyde-3-phosphate dehydrogenase (GAPDH). The sequences of the primers used in this study are listed in Table S2.

### RNA-sequencing analysis

A total of 12 samples were used to ensure that the number of cells in each sample was 1 × 10^6^ and were divided into 4 groups: control, UVB, UVB/CS, and UVB/ZnCS (*n* = 3 per group). Total RNA was extracted using a TRIzol-based method, and its quality was verified on an Agilent 2100 Bioanalyzer. Poly(A) mRNA was enriched using oligo(dT) magnetic beads, purified, and chemically fragmented with a fragmentation buffer. Sequencing libraries were constructed following Illumina’s protocol and sequenced on an Illumina NovaSeq 6000 with paired-end 150–base pair reads. Raw reads were quality-checked (using FastQC and Trimmomatic), aligned to the reference genome with HISAT2, and quantified using featureCounts; normalization was performed with DESeq2. Differential expression analysis was conducted with DESeq2, with DEGs (differentially expressed genes) defined as those showing a fold change >2 and an adjusted *P* value < 0.05. GO enrichment analysis was carried out using the clusterProfiler package (v3.10.1) with annotations from the Gene Ontology Consortium, employing a hypergeometric test with Benjamini–Hochberg correction. Kyoto Encyclopedia of Genes and Genomes (KEGG) pathway analysis was performed using the KEGG database (https://www.genome.jp/kegg/pathway.html) to functionally annotate DEGs. The enrichment results were visualized using R language, specifically with the enrichplot and DOSE packages, to generate plots that illustrate the biological pathways and processes involved.

### ABTS assay

The Trolox equivalent antioxidant capacity (TEAC) is used to express the antioxidant capacity of a sample. The ABTS [2,2'-azino-bis(3-ethylbenzothiazoline-6-sulfonic acid)] assay was conducted as described in the Total Antioxidant Capacity Assay Kit (A015-2-1, Nanjing Bioengineering Research Institute, Jianjian). The cell samples were collected, and the protein concentration was determined via the BCA (bicinchoninic acid) method. The standard Trolox solution was diluted to concentrations of 0.1, 0.2, 0.4, 0.8, and 1.0 mM with distilled water to generate a standard curve, and the application solution and ABTS working solution were prepared and added. Then, the reaction was carried out at room temperature for 6 min at a wavelength of 405 nm, and the OD (optical density) value of each well was read by an enzyme counter.

### Western blot analysis

NIH-3T3 cells and skin tissue were lysed via tris-based protein lysis buffer on ice for 1 h. After centrifugation (12,000 rpm, 4 °C, 15 min), the supernatants were subjected to Western blotting. Twenty micrograms of lysate was electrophoresed in a sodium dodecyl sulfate (SDS) polyacrylamide gel and transferred to nitrocellulose membranes. After the nonspecific signals were blocked by incubation with blocking buffer, the membranes were incubated with each primary antibody (1:1,000) for 12 to 14 h at 4 °C. Protein expression was assessed via the following primary antibodies: PKA-Cα (protein kinase A catalytic subunit α) (1:1,000; Cell Signaling Technology, catalog no. 5842), CREB (cAMP response element-binding protein) (1:1,000; Cell Signaling Technology, catalog no. 9197), phosphatidylinositol 3-kinase (PI3K) (1:1,000; Cell Signaling Technology, catalog no. 49), Akt (1:1,000; Cell Signaling Technology, catalog no. 9272), and β-actin (1:1,000; Cell Signaling Technology, catalog no. 4967). After the samples were washed with tris-buffered saline with Tween (TBS-T), horseradish peroxidase (HRP)-conjugated secondary antibody (1:3,000) was added, and the samples were incubated at room temperature for 2 h. Protein bands were quantified via densitometry using Image Lab Software v6.1 (Bio-Rad). Membranes were imaged with a ChemiDoc MP System (Bio-Rad) under auto-exposure settings, and background subtraction was applied uniformly. Target protein levels were normalized to β-actin expression within the same lane. Three technical replicates per sample were averaged to minimize variability. Normalized data were expressed as fold change relative to the control group using the ΔΔ*C*_t_ method.

### Statistical analyses

The statistical analyses were performed via GraphPad Prism 7.0 (GraphPad, CA, USA). The data obtained from different samples are presented as the mean ± standard error of the mean (SEM). One-way analysis of variance (ANOVA) was performed, followed by Tukey’s HSD (honest significant difference) for multiple comparisons. A value of *P < 0.05, **P < 0.01, ***P < 0.001, or ****P < 0.0001 was considered to indicate statistical significance.

## Results

### Characterization of hardystonite (ZnCS) bioceramics and composite MNs (ZnCS/MN)

First, we successfully synthesized hardystonite (ZnCS) and CS (used as a control group) bioceramics. The SEM image illustrates the irregular shapes of both bioceramics (Fig. S1A). Notably, EDS analysis revealed consistent enrichment of Ca, Si, and O in the CS bioceramics, whereas the ZnCS bioceramics exhibited uniform enrichment of Ca, Si, O, and Zn (Fig. S1B).

ZnCS and CS were subsequently used to prepare composite microneedles. Optical photos revealed that sodium hyaluronate microneedles (Blank) are colorless and transparent, whereas microneedles loaded with CS (CS/MN) or hardystonite (ZnCS/MN) bioceramics are white (Fig. 1A). SEM images show arranged arrays for each microneedle group, featuring a tip height of approximately 600 μm, which is high enough to penetrate the skin epidermal layer (approximately 50 μm) [26]. Additionally, the surface roughness of the microneedles loaded with CS or ZnCS bioceramics was notably greater than that of the pure sodium hyaluronate microneedle group, particularly in terms of the visible enrichment of bioceramic particles on the needle tips of the composite microneedles (Fig. 1B). Moreover, on the first day, the concentration of SiO_3_^2−^ ions released from ZnCS/MN was 6.51 ± 1.19 μg/ml. This concentration steadily decreased to 1.93 ± 0.52 μg/ml by the fifth day. Notably, there was no significant difference in the SiO_3_^2−^ concentration between ZnCS/MN and CS/MN. Furthermore, ZnCS/MN exhibited a sustained release of Zn^2+^ ions, with an initial concentration of 2.01 ± 0.43 μg/ml on the first day, which gradually decreased to 0.71 ± 0.14 μg/ml by the fifth day (Fig. 1C).

### Effect of ZnCS extract on promoting the viability of photodamaged fibroblasts

We evaluated the viability of fibroblasts treated with the extracts of ZnCS and CS (used as a control group). Interestingly, we observed that the ZnCS extract, with dilutions ranging from 1:4 to 1:128, and the CS extract, with dilutions ranging from 1:2 to 1:128, exhibited no cytotoxicity. Notably, at a dilution ratio of 1:32, both solutions resulted in the most significant increase in fibroblast viability (Figs. S2 and S3). At this dilution ratio, the concentration of SiO_3_^2−^ ions in the CS extract was 4.06 ± 0.27 μg/ml, the concentration of SiO_3_^2−^ ions in the ZnCS extract was 5.13 ± 0.05 μg/ml, and the Zn^2+^ concentration was 0.96 ± 0.06 μg/ml (Table S1), which are within the range of bioactive concentrations of Zn^2+^ and SiO_3_^2−^ reported in previous experiments [24,26–28]. Therefore, we selected a bioceramic extract with a dilution of 1:32 for subsequent studies.

**Fig. 2.**
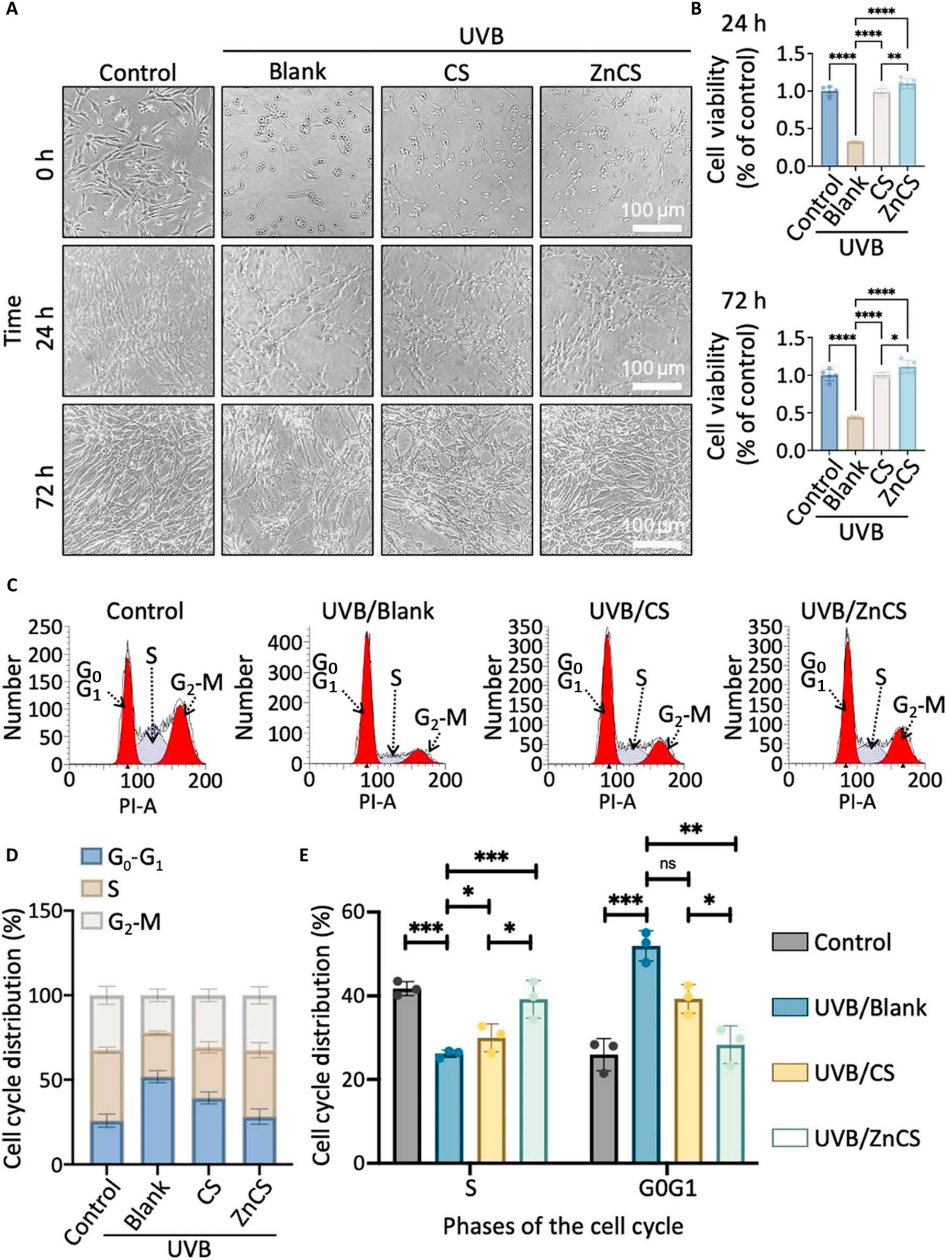
Impact of ZnCS on the viability and cell cycle of photodamaged fibroblasts. (A) Microscopic images of photodamaged fibroblasts at 0, 24, and 72 h treated with ZnCS extract. (B) Cell viability of photodamaged fibroblasts via CCK-8 assay at 0 and 72 h treated with ZnCS extract (*n* = 4). (C) Flow cytometric analysis of the cell cycle of photodamaged fibroblasts treated with ZnCS extract at 48 h. (D) Statistical analysis of the distribution of cell cycles of photodamaged fibroblasts. (E) Statistical analysis of the percentage of S phase and G_0_-G_1_ phase of photodamaged fibroblasts. Experiments were conducted a minimum of 3 times for accuracy. *P < 0.05, **P < 0.01, ***P < 0.001, ****P < 0.0001, and ns: no significance. Control: Fibroblasts cultured in normal cell cultured medium; UVB/Blank: Photodamaged fibroblasts cultured in normal cell cultured medium; UVB/CS: Photodamaged fibroblasts cultured in CS extract; UVB/ZnCS: Photodamaged fibroblasts cultured in ZnCS extract; CS extract dilution ratio: 1:32; ZnCS extract dilution ratio: 1:32.

To investigate the effects of ZnCS on photodamage, fibroblasts were exposed to UVB radiation at an intensity of 120 mJ/cm^2^ to create an in vitro UVB-induced damage model. Immediately following irradiation (0 h), the fibroblasts exhibited morphological changes characterized by shrinkage under microscopic observation (Fig. 2A). Notably, after culturing for 24 and 72 h, the viability of the cells treated with the ZnCS and CS extracts was comparable to that of the control cells and significantly greater than that of the UVB-induced cells (UVB/Blank), as determined via a CCK-8 assay (Fig. 2B). Moreover, compared with CS, ZnCS more effectively enhanced fibroblast viability (Fig. 2B).

Additionally, flow cytometry was used to analyze the cell cycle distribution of the fibroblasts. Among UVB-induced cells (Blank), there was a notable increase in the percentage of cells in the G_0_-G_1_ (gap/interphase) phase and a decrease in the percentage of cells in the S (synthesis) phase compared with those in the control group (Fig. 2C and D), indicating that UVB exposure inhibits fibroblast proliferation [29]. In contrast, both the ZnCS- and CS extract-treated groups presented a significantly greater percentage of S-phase cells and a lower percentage of G_0_-G_1_-phase cells, and in comparison with the CS group, the ZnCS group presented a greater percentage of S-phase cells (Fig. 2E).

### Effect of ZnCS on the inhibition of ROS and inflammation in UVB-induced fibroblasts

**Fig. 3.**
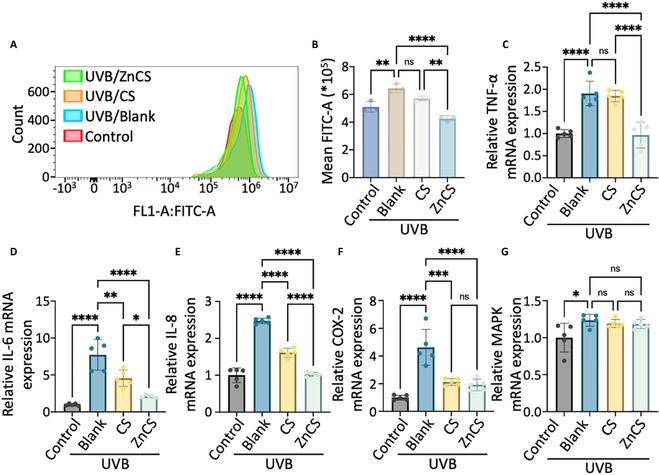
Evaluation of the inhibitory impact of hardystonite (ZnCS) extract on ROS and inflammation in photodamaged fibroblasts (after UVB irradiation for 48 h). (A) Number of photodamaged fibroblasts producing ROS. (B) Statistical analysis of fluorescence intensity of produced ROS in photodamaged fibroblasts (*n* = 3). (C to G) RT-qPCR results illustrating the relative mRNA expression of TNF-α, IL-6, IL-8, COX-2, and MAPK (*n* = 5). *P < 0.05, **P < 0.01, ***P < 0.001, ****P < 0.0001, and ns: no significance. Control: Fibroblasts cultured in normal cell cultured medium; UVB/Blank: Photodamaged fibroblasts cultured in normal cell cultured medium; UVB/CS (calcium silicate): photodamaged fibroblasts cultured in CS extract; UVB/ZnCS: Photodamaged fibroblasts cultured in ZnCS extract; CS extract dilution ratio: 1:32; ZnCS extract dilution ratio: 1:32.

The accumulation of ROS within cells is one of the main key factors for UVB-induced photodamaged skin inflammation [4]. Therefore, the intracellular ROS levels were first assessed via flow cytometry with a dihydroethidium (DHE) probe. The results revealed that UVB-irradiated fibroblasts produced more ROS than cells not treated with UVB (Fig. 3A and B). Remarkably, a significant reduction in ROS was observed in both the ZnCS and CS groups, especially in the ZnCS group (Fig. 3A and B). Furthermore, we utilized quantitative reverse transcription PCR (qRT-PCR) to assess the expression of inflammatory factors [tumor necrosis factor-α (TNF-α), interleukin-6 (IL-6), IL-8, cyclooxygenase-2 (COX-2), and mitogen-activated protein kinase (MAPK)] in fibroblasts from each group. The results indicated that both ZnCS and CS significantly inhibited the expression of TNF-α, IL-6, IL-8, and COX-2. Notably, the ZnCS treatment had a significantly greater inhibitory effect on IL-6 and COX-2 than the CS treatment (Fig. 3C to G).

### Effect of ZnCS/MN on repairing UVB-induced photodamaged skin

**Fig. 4.**
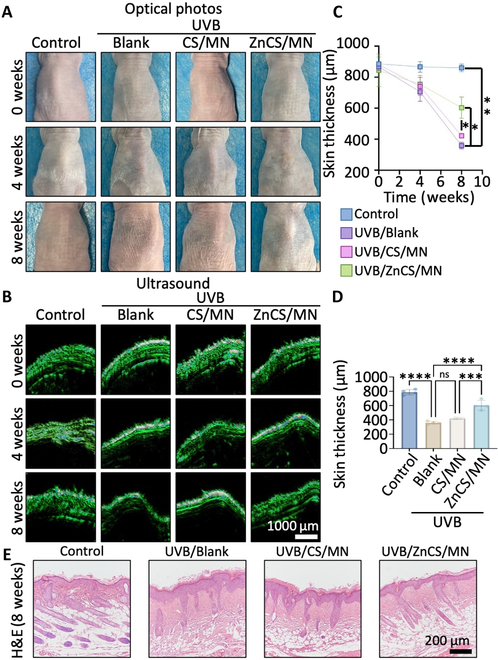
Detection of the skin of photodamaged mice treated with ZnCS/MN. (A) Skin appearance of mice in each group at weeks 0, 4, and 8. (B) Skin ultrasonography images of mice at weeks 0, 4, and 8. (C) Skin thickness of mice in each group at weeks 0, 4, and 8 according to the ultrasonography images. (D) H&E staining images of mice skin. (E) Skin thickness of mice in each group at week 8 according to the H&E staining images. *P < 0.05, **P < 0.01 ***P < 0.001, ****P < 0.0001, and ns: no significance. Control: Healthy mice; UVB/Blank: Photodamaged mice treated with MN; UVB/CS/MN: Photodamaged mice treated with CS/MN; UVB/ZnCS/MN: photodamaged mice treated with ZnCS/MN.

To investigate the ability of ZnCS/MN to repair photodamaged skin, an in vivo skin photodamage model was created through chronic UVB radiation, and the mice were treated with MN (UVB/Blank), CS/MN (UVB/CS/MN), or ZnCS/MN (UVB/ZnCS/MN) (Fig. S4). Optical photos revealed significant skin shrinkage in the UVB/Blank group compared with the control group by the eighth week. Interestingly, the mice treated with ZnCS/MN had a smoother skin surface, whereas the CS/MN did not seem to have a significant effect on preventing skin wrinkles (Fig. 4A). Further skin ultrasonography revealed that the skin thickness of the mice in the ZnCS/MN group was greater than that of the UVB/Blank and UVB/CS/MN groups at week 8, whereas the skin thickness did not significantly differ at week 4 after UVB irradiation (Fig. 4B and C). Moreover, H&E-stained tissue sections were used to observe the skin thickness of each group at 8 weeks. Histological analysis confirmed the ultrasonography results that the skin thickness in the ZnCS/MN group was indeed significantly greater than that in the Blank and CS/MN groups and was close to the thickness of normal skin (control) (Fig. 4D). Additionally, H&E staining revealed hair follicle tissues with a normal morphology in the back skin of ZnCS/MN-treated mice (Fig. 4E and Fig. S5). Notably, ZnCS/MN significantly reduced UVB-induced senescence. This was evidenced by the substantial inhibition of SA-β-Gal (senescence-associated β-galactosidase) and P21, which are typical markers of cell senescence (Figs. S6 to S8).

### Effect of ZnCS/MN on the inhibition of ROS production, inflammation, and collagen degradation

**Fig. 5.**
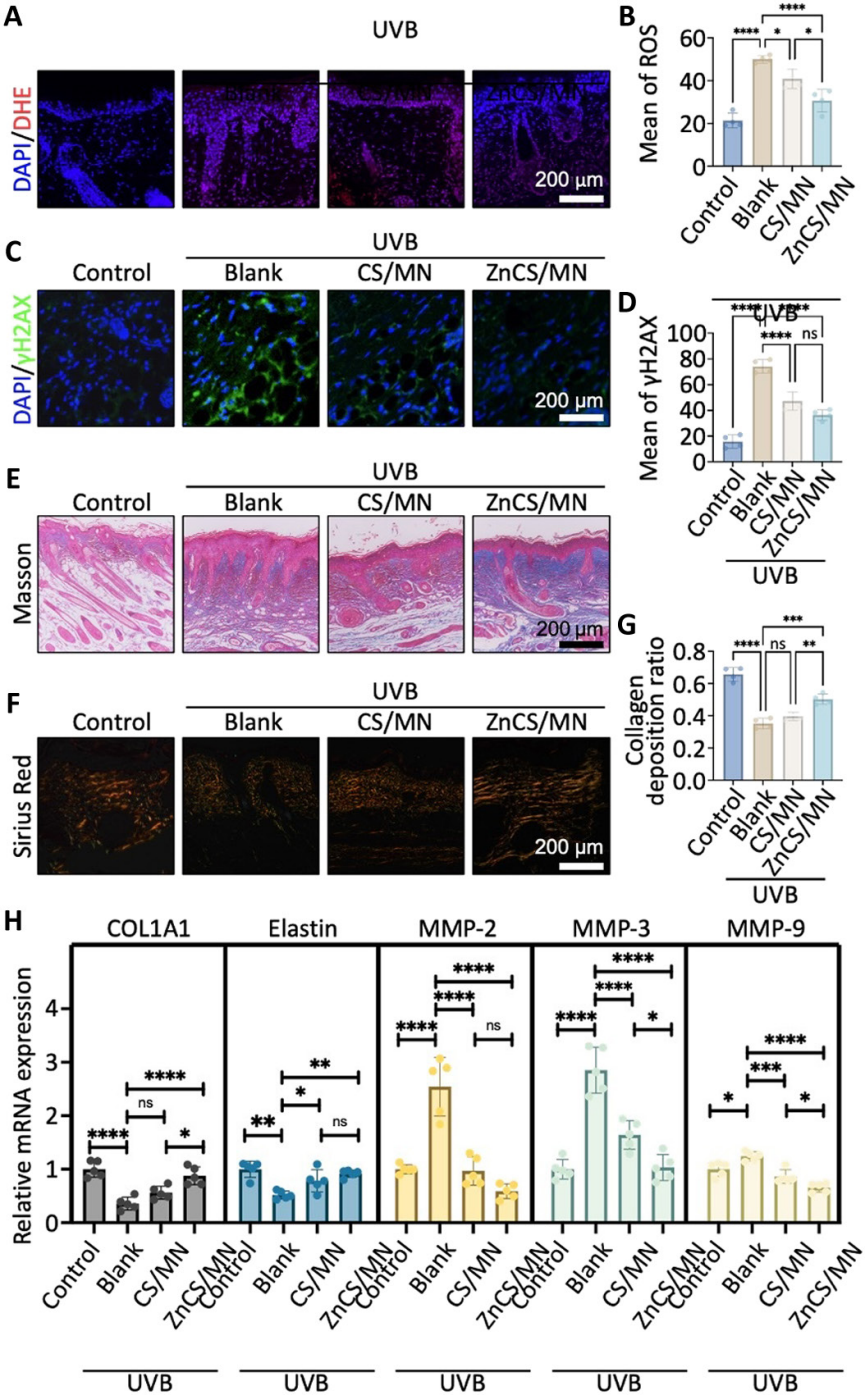
Detection of ROS, inflammation, and collagen changes in photodamaged mice skin treated with ZnCS/MN. (A) ROS production (DHE staining) in photodamaged mice skin at week 8 (DHE: red, DAPI: blue). (B) Statistical analysis of ROS production (*n* = 3). (C) Inflammation-related DNA damage (γH2AX staining) in photodamaged mice skin at week 8 (γH2AX: green; DAPI: blue). (D) Statistical analysis of the relative fluorescence intensity of γH2AX (*n* = 4). (E and F) Masson staining and Sirius red staining (polarized light microscopy) of photodamaged mice skin at week 8 (Masson: collagen in blue, Sirius Red: collagen I in red, collagen III in green). (G) Statistical analysis of the collagen I deposition ratio [collagen I/(collagen I + collagen III)] according to Sirius Red staining results (*n* = 4). (H) Relative mRNA expression of COL1A1, elastin, MMP-2, MMP-3, and MMP-9 by RT-qPCR (*n* = 5). *P < 0.05, **P < 0.01 ***P < 0.001, ****P < 0.0001, and ns: no significance. Control: Healthy mice; UVB/Blank: Photodamaged mice treated with MN; UVB/CS/MN: Photodamaged mice treated with CS/MN; UVB/ZnCS/MN: Photodamaged mice treated with ZnCS/MN.

To validate the inhibitory effects of ZnCS/MNs on ROS production and inflammation, immunofluorescence staining for DHE (a ROS marker) and γH2AX (an inflammatory marker indicative of DNA damage caused by inflammation) was conducted on UVB-induced photodamaged mouse skin. The fluorescence images clearly demonstrated that, compared with those in the UVB/Blank group, the accumulation of ROS in the skin was significantly lower in the ZnCS/MN group, whereas the expression of DHE (red fluorescence) was only slightly greater in the ZnCS/MN group than in the control group (Fig. 5A). Quantitative analysis further revealed that CS/MN moderately inhibited ROS accumulation, whereas ZnCS/MN significantly inhibited ROS accumulation in UVB-induced photodamaged skin (Fig. 5B). Additionally, the γH2AX immunofluorescence images revealed that the ZnCS/MN combination most significantly inhibited the expression of γH2AX (Fig. 5C). Notably, compared with those in the UVB/Blank group, both ZnCS/MN and CS/MN significantly inhibited inflammation-related DNA damage, and the effects of the 2 were comparable (Fig. 5D). Furthermore, acute photodamage models were constructed through high-dose UVB irradiation (800 mJ/cm^2^), and the significant inhibitory effects of ZnCS/MN on ROS and inflammation were further confirmed (Fig. S9).

The efficacy of ZnCS/MN in inhibiting collagen degradation was evaluated via the use of collagen I, which is the typical collagen type in the dermis and is responsible for the tensile strength of skin tissue, and its synthesis is often hindered after UVB irradiation [15,30]. Masson and Sirius Red staining revealed that, in comparison with those in UVB/Blank and UVB/CS/MN mice, the skin of mice treated with ZnCS/MN presented more abundant collagen, particularly collagen I (Masson: collagen in blue, Sirius Red: collagen I in red, collagen III in green) (Fig. 5E and F). Quantitative statistical analysis of type I/(I + III) collagen in Sirius Red-stained sections revealed that ZnCS/MN intervention significantly increased the proportion of type I collagen (Fig. 5G). Additionally, the qRT-PCR results revealed that ZnCS/MN treatment significantly promoted the expression of COL1A1 and elastin, whereas CS/MN treatment increased only the expression of elastin (Fig. 5H). Considering that matrix metalloproteinases (MMPs) play crucial roles in collagen degradation in UVB-induced photodamaged skin [15], we conducted a detailed assessment of the expression levels of MMP-2, MMP-3, and MMP-9. The results indicated that both the ZnCS/MN and CS/MN treatments significantly decreased the expression levels of MMP-2, MMP-3, and MMP-9. Notably, compared with CS/MN, ZnCS/MN more strongly inhibited MMP-3 and MMP-9 (Fig. 5H).

### Biological mechanism of ZnCS in treating skin photodegradation

**Fig. 6.**
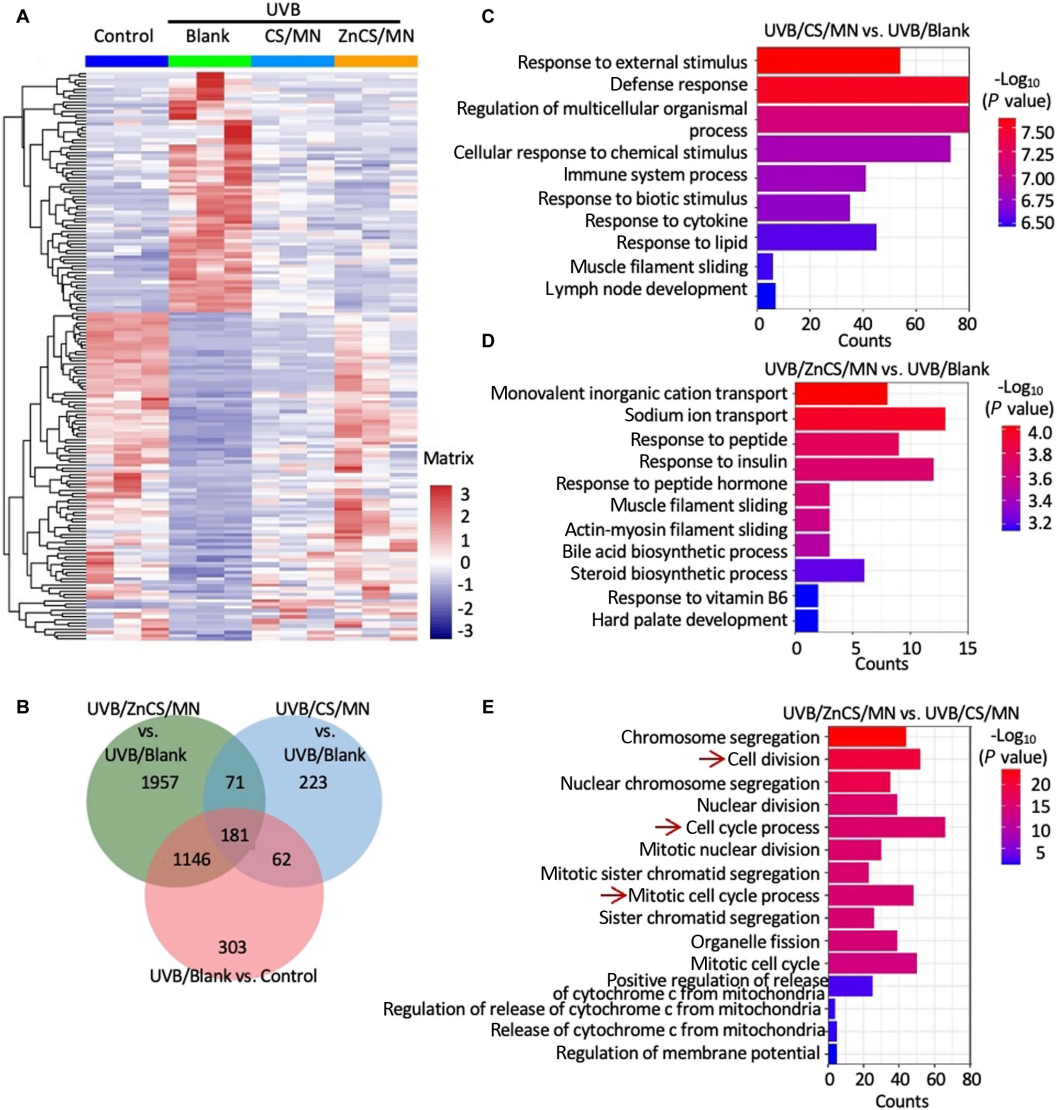
Differential gene expression and enrichment after ZnCS intervention. (A) Heatmap showing the differential genes in the Control, UVB/Blank/MN, UVB/CS/MN, and UVB/ZnCS/MN groups. (B) Venn diagram. (C) Up-regulated biological processes in GO analysis for the UVB/ZnCS/MN group compared to the UVB/Blank/MN group. (D) Up-regulated biological processes in GO analysis for the UVB/CS/MN group compared to the UVB/Blank/MN group. (E) Up-regulated biological processes in GO analysis for the UVB/ZnCS/MN groups compared to the UVB/CS/MN group. (A to E) UVB/Blank: Photodamaged mice treated with MN; UVB/CS/MN: Photodamaged mice treated with CS/MN; UVB/ZnCS/MN: Photodamaged mice treated with ZnCS/MN.

To determine the mechanism by which ZnCS/MN affects UVB-induced photodamage, we conducted RNA sequencing and evaluated gene expression patterns in photodamaged mouse skin (Fig. 6A and Fig. S10). A heatmap was generated to visualize the DEGs among the 4 experimental groups (Fig. 6A). The gene expression patterns of the UVB/ZnCS/MN group were more similar to those of the control group (Fig. 6A). We also performed Venn diagram analysis to identify the overlapping DEGs among the 3 comparisons (UVB/CS/MN versus UVB/Blank, UVB/ZnCS/MN versus UVB/Blank, and UVB/ZnCS/MN versus UVB/CS/MN), and UVB/ZnCS/MN presented more DEGs than UVB/CS/MN did (Fig. 6B and Fig. S11). Gene Ontology (GO) analysis revealed significant enrichment in terms of up-regulated “biological processes” related to cell proliferation, such as cell cycle processes, cell division, and mitotic nuclear division, in the UVB/ZnCS/MN group (Fig. 6E).

**Fig. 7.**
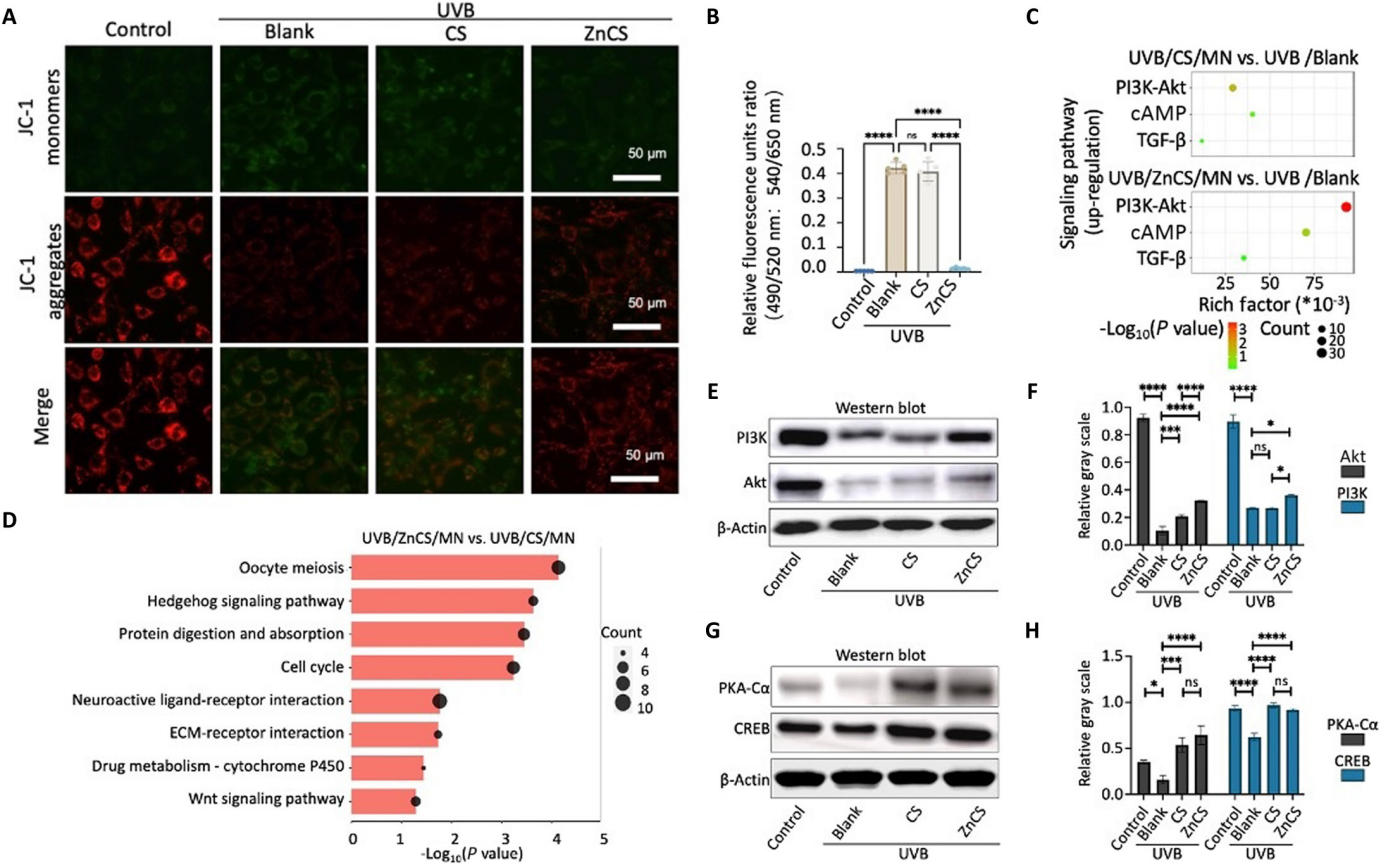
The multiple mechanisms of ZnCS in reducing skin photodamage. (A and B) Images and relative fluorescence intensity of mitochondrial membrane potential (MMP) of JC-2 aggregates and monomers from 4 groups. (C) Up-regulated pathways in KEGG analysis for the UVB + CS and UVB + ZnCS groups. (D) Up-regulated pathways in KEGG analysis for the UVB/ZnCS/MN groups compared to the UVB/CS/MN group. (E) Western blotting results of PI3K and Akt expression. (F) Statistical analysis of Western blotting results for PI3K and Akt expression (*n* = 3). (G) Western blotting results of PKA and CREB expression. (H) Statistical analysis of Western blotting results for PKA and CREB expression (*n* = 3). *P < 0.05, **P < 0.01 ***P < 0.001, ****P < 0.0001, and ns: no significance. (C and D) UVB/Blank: Photodamaged mice treated with MN; UVB/CS/MN: Photodamaged mice treated with CS/MN; UVB/ZnCS/MN: Photodamaged mice treated with ZnCS/MN. (A, B, and E to H) Control: Fibroblasts cultured in normal cell cultured medium; UVB/Blank: Photodamaged fibroblasts cultured in normal cell cultured medium; UVB/CS: Photodamaged fibroblasts cultured in CS extract; UVB/ZnCS: Photodamaged fibroblasts cultured in ZnCS extract; CS extract dilution ratio: 1:32; ZnCS extract dilution ratio: 1:32.

**Fig. 8.**
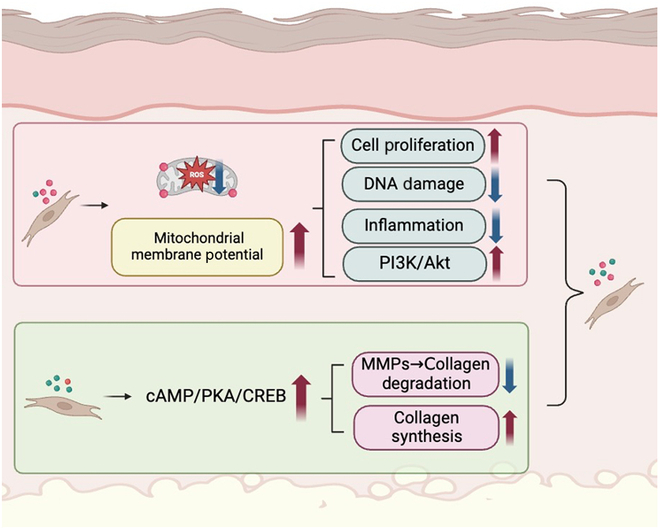
The mechanism of ZnCS/MN in treating UVB-induced skin photodamage. Zn^2+^ primarily stabilized mitochondrial membrane potential to modulate Akt/PI3K, reduce inflammation, promote cell proliferation, and inhibit DNA damage (with synergistic assistance from SiO_3_^2−^). Concurrently, SiO_3_^2−^ ions primarily regulate cAMP/PKA/CREB to inhibit MMPs related collagen degradation and enhance collagen synthesis (with synergistic assistance from Zn^2+^).

Next, we investigated the mechanism underlying the beneficial effect of ZnCSs on ROS inhibition. In vitro, UVB-induced fibroblasts presented a decreased mitochondrial membrane potential, whereas ZnCS extract increased the mitochondrial membrane potential in UVB-induced fibroblasts (Fig. 7A and B) and improved the antioxidant capacity (Fig. S12). Moreover, in the GO analysis of the UVB/ZnCS/MN versus UVB/CS/MN comparisons, we identified enrichment in the terms “membrane potential” and “cytochrome c”, which are associated with mitochondrial function (Fig. 7D). KEGG pathway analysis revealed significant up-regulation of the PI3K/Akt pathway, an anti-inflammatory pathway that is inhibited by excessive ROS [31], in the skin of plants treated with UVB/ZnCS/MN compared with that in the skin of UVB/CS/MN mice (Fig. 7E and G and Fig. S13). Furthermore, we found that the cAMP pathway was up-regulated in the UVB/CS/MN group, which may account for the inhibitory effect of CS on ROS production (Fig. 7C, E, and H). We subsequently cultured UVB-induced photodamaged fibroblasts in the presence of ZnCS extract and analyzed protein secretion via Western blotting. Notably, ZnCS significantly increased PI3K and Akt expression (Fig. 7E and F and Fig. S14). Additionally, the expression of PKA-Cα and CREB in UVB-induced fibroblasts treated with CS or ZnCS was greater than that in UVB-treated fibroblasts (Fig. 7G and H). After treatment with an inhibitor of cAMP (SQ22536), the inhibition of MMP expression in the CS group was reduced (Fig. S15), suggesting that SiO_3_^2−^ promoted collagen synthesis and inhibited degradation mainly via the cAMP/PKA/CREB pathway. These findings indicated that ZnCS promoted cell proliferation, exerting anti-inflammatory effects and enhancing collagen synthesis through the stabilization of the mitochondrial membrane potential and up-regulation of the cAMP pathway, leading to the suppression of ROS production (Fig. 8).

## Discussion

UVR-related skin damage directly inhibits the synthesis of collagen in skin fibroblasts and promotes collagen degradation, subsequently causing skin aging, wrinkles, and erythema [32,33]. Prolonged UVB exposure also increases the risk of skin cell damage, inducing photosensitive skin diseases and skin malignancies [34]. Current clinical treatments, such as retinoid application and laser therapy, provide only partial relief and often come with side effects, highlighting the need for alternative approaches [51, 52]**.** Considering that application to the surface cannot penetrate the skin’s epidermal barrier [35–37], we designed a sodium hyaluronate microneedle patch containing a hardystonite bioceramic (ZnCS/MN) and facilitated the direct delivery of bioactive Zn^2+^ and SiO_3_^2−^ ions in situ to photodamaged skin areas [38,39]. According to our results, the skin structure of the mice treated with ZnCS/MN more closely resembled that of normal skin. The level of ROS and the expression of inflammatory factors were significantly decreased in the UVB/ZnCS group. These findings are consistent with prior studies demonstrating that Zn^2+^ plays a key role in antioxidant defense mechanisms by stabilizing cellular redox homeostasis and enhancing mitochondrial function [25,27]. UVB exposure leads to the production of ROS in the skin, resulting in inflammation [40,41]. Excessive ROS, which lead to alterations in the cellular DNA structure and decreased cell capacity, as well as an intracellular inflammatory response, are the primary contributors to skin photodamage [42]. The destabilization of the mitochondrial membrane potential due to UVB irradiation is a significant factor contributing to elevated ROS levels [43,44]. Our findings demonstrated that ZnCS intervention stabilized the mitochondrial membrane potential. Moreover, GO analysis revealed the up-regulation of cytochrome and membrane potential release, in agreement with the finding that ZnCS stabilized the mitochondrial membrane potential.

DNA damage, inflammation, and cell senescence, which are consequences of accumulated oxidative stress, were alleviated in the UVB/ZnCS group. Compared with the CS group, the ZnCS group was enriched in cell cycle-related pathways, and the inhibition of inflammatory factors (TNF-α, IL-6, and IL-8) was more significant. Excessive accumulation of ROS leads to suppression of the PI3K/Akt pathway, ultimately resulting in DNA damage, inflammatory responses, and cell cycle arrest [31]. By suppressing ROS production, ZnCS up-regulated the PI3K/Akt signaling pathway and exerted significant effects on protecting DNA integrity, facilitating cell proliferation, and reinforcing anti-inflammatory responses. ZnCS had a greater inhibitory effect on ROS, which is an important reason why ZnCS had better effects on photodamaged mouse skin than CS.

These findings align with existing literature on silicate-based biomaterials, which have been shown to modulate oxidative stress and promote tissue regeneration via bioactive ion release [16,17]. However, our study extends this knowledge by demonstrating that ZnCS not only mitigates oxidative stress but also exerts direct effects on mitochondrial stability and cell cycle regulation. This suggests a more comprehensive mechanism by which ZnCS promotes skin repair beyond simple ROS neutralization.

Moreover, CS contributed to a reduction in the levels of ROS and inflammatory factors. Our results revealed enrichment of the cAMP/PKA/CREB pathway in the groups treated with CS and ZnCS. cAMP is an important second messenger and is closely related to the function of mitochondria. An increase in cAMP was reported to inhibit ROS overproduction in oxidatively damaged cells [45]. cAMP activates CREB in mitochondria, which induces the expression of OXPHOS (oxidative phosphorylation) subunits and leads to a decrease in ROS production [45]. Previous studies have highlighted the role of cAMP/PKA/CREB signaling in cellular metabolism and redox regulation; our findings further establish its relevance in skin photodamage repair, particularly in the modulation of MMP expression and collagen degradation [15,30]. Therefore, ZnCS has a superior inhibitory effect on ROS production through the synergistic action of Zn^2+^ in stabilizing the mitochondrial membrane potential and SiO_3_^2−^ in the cAMP pathway.

UVB-exposed fibroblasts exhibit decreased collagen synthesis capacity and up-regulated MMP expression, leading to collagen degradation [15]. The cAMP/PKA/CREB pathway has been reported to be the principal signaling pathway responsible for regulating matrix metalloproteinases (MMPs, enzymes implicated in collagen degradation), but its role in skin photodamage has not been investigated before [30,46]. In our study, we found that the ability of SiO_3_^2−^ to down-regulate the MMP was suppressed by a cAMP inhibitor. This suggests that the protective effects of ZnCS on collagen integrity are at least partially mediated through cAMP/PKA/CREB signaling, providing a mechanistic basis for its superior efficacy in preventing UVB-induced collagen degradation compared to conventional treatments. These results were consistent with the fact that ZnCS and CS directly inhibited the expression of MMPs to attenuate collagen degradation in UVB-exposed mouse skin. Additionally, ZnCS promoted the mRNA expression of COL1A1, which was also due to the high bioactivity of ZnCS in lowering ROS levels, thus minimizing relative cell damage through the synergistic effect of these 2 ions. Therefore, the skin of the mice in the ZnCS/MN group was thicker than that in the UVB/Blank group, and the ZnCS/MN group had a greater collagen content and ratio of collagen I than the other groups, suggesting that Zn^2+^ in combination with SiO_3_^2−^ had the most significant protective effect on preventing UVB-induced skin damage.

ZnCS shows desirable therapeutic efficacy in various aspects of photodamage. Compared to traditional microneedle-based therapies, ZnCS/MN demonstrates a more targeted and prolonged release of bioactive ions, enhancing transdermal drug delivery. Nevertheless, ZnCS/MN has clinical potential for photodamaged skin treatment by reducing oxidative stress preventing collagen degradation. As opposed to standard clinical therapies like topical retinoids and clinical procedures (e.g., chemical peels and lasers) , ZnCS/MN offers a more precise delivery without the discomfort or side effects such as irritation or high cost. While retinoids can improve skin texture with long-term use, they require daily application, and clinical treatments often involve high expenses and risks like pigmentation or erythema. Antioxidants and stem cell therapies exhibit restricted efficacy or safety concerns [12]. Moreover, as an inorganic ion, ZnCS is more stable, making it well-suited for large-scale production and long-term storage even at room temperature. .

In addition, we explored the differences between ZnCS and CS, with ZnCS being superior in inhibiting ROS production and improving skin collagen structure and CS having an additional role in inhibiting collagen degradation through the cAMP pathway. These findings provide critical insights into the differential bioactivities of Zn^2+^ and SiO₃^2−^, suggesting that tailored combinations of bioactive ions could further optimize therapeutic outcomes in skin regeneration. Owing to its advantages in terms of preparation, storage, and transportation, ZnCS/MN has shown promise for clinical application in treating photodamaging [35–39]. The mechanism through which ZnCS treats photodamaged skin needs to be investigated in greater depth. The mechanism through which Zn^2+^ stabilizes the mitochondrial membrane potential requires further investigation [47]. Moreover, although ZnCS/MN demonstrated good effects in mouse models, its effectiveness must be validated in clinical studies in the future.

## Conclusion

Our study demonstrated that ZnCS/MN is an ideal approach for the treatment of photodamaged skin. Zn^2+^ and SiO_3_^2−^ had synergistic effects on reducing ROS production, as Zn stabilized the mitochondrial membrane potential, and SiO_3_^2−^ up-regulated the cAMP/PKA/CREB pathway, leading to anti-inflammatory, proliferation-enhancing, and DNA-protective effects. Moreover, SiO_3_^2−^ can not only promote collagen synthesis but also simultaneously reduce collagen degradation via the cAMP/PKA/CREB pathway. After ZnCS/MN treatment, the thickness and collagen content of UVB-induced skin in the dermis improved. Overall, the desirable therapeutic effects of ZnCS/MN in photodamaged skin repair, as well as the advantages of easy fabrication and low cost, have value for further research and clinical translation potential in the future.

## Data Availability

Data are available within the article or its Supplementary Materials. More data that support the findings of this study are available from the corresponding author, S.Z., upon reasonable request.
