# Supplementary material for: Zinc Silicate-Loaded Microneedle Patch Reduces Reactive Oxygen Species Production and Enhances Collagen Synthesis for Ultraviolet B-Induced Skin Repair
Source: Biomater Res. 2025 Apr 10;29:0180. doi: 10.34133/bmr.0180 (PMC11982614; doi:10.34133/bmr.0180)
Supplement: Supplementary 1 — Figs. S1 to S15 Tables S1 to S3 [file bmr.0180.f1.docx]

**Supporting Information**


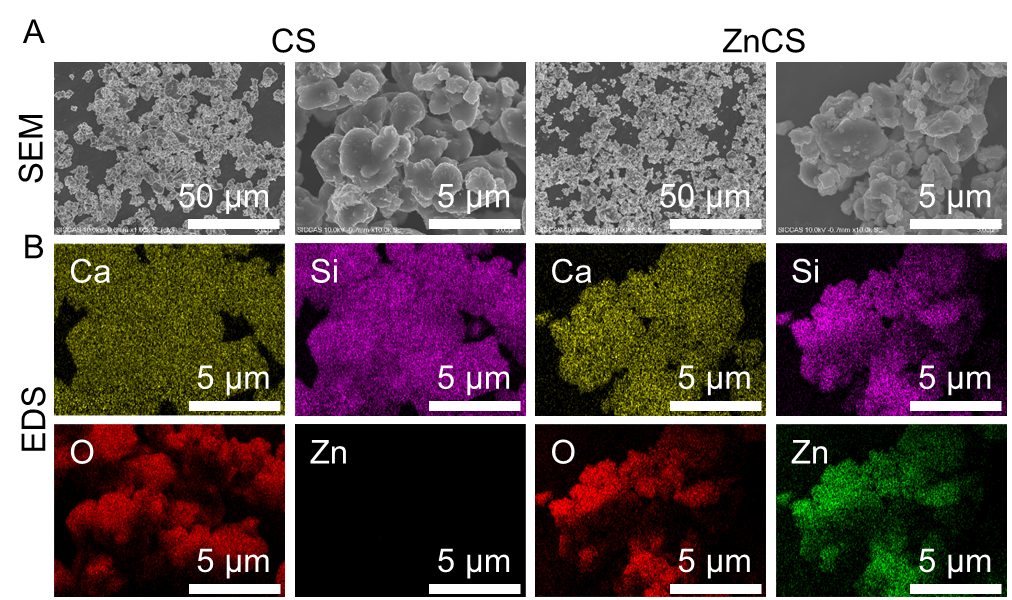


**Figure S1.** Scanning electron microscopy (SEM) and Elemental distribution were employed to characterize the morphology and crystalline structure of Calcium silicate (CS) and Hardystonite (ZnCS) bioceramics. (A) SEM images of the CS and ZnCS bioceramics. (B) Elemental distribution of the CS and ZnCS bioceramics.

**Figure S2.** Effects of CS extract on the viability of fibroblasts. *P < 0.05.

**Figure S3.** Effects of ZnCS extract on the viability of fibroblasts. *p < 0.05.


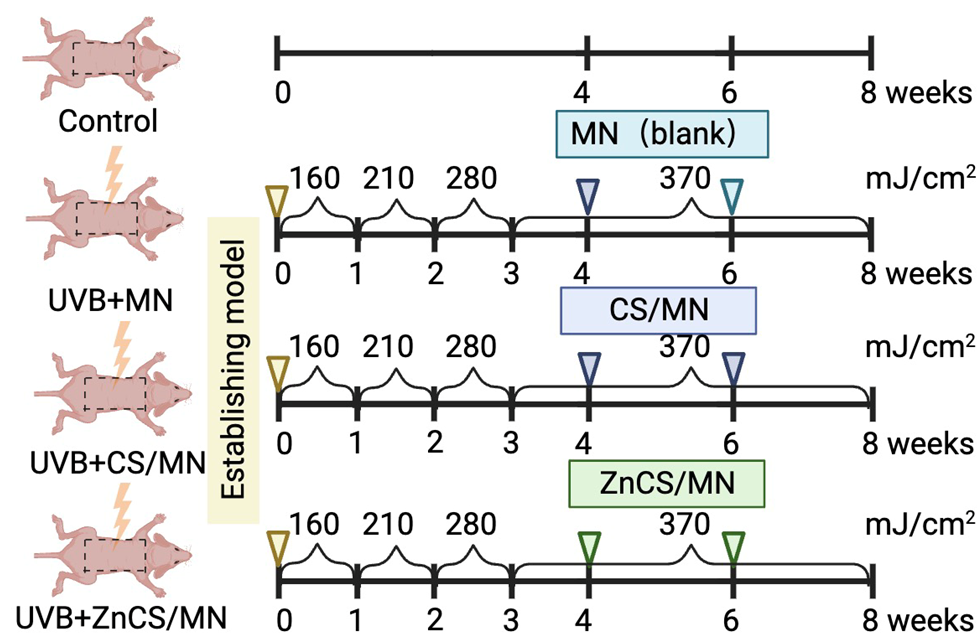


**Figure S4.** Establishment of a mouse model of photodamaged by UVB and treatment with ZnCS/MN.

Control: Healthy mice;

UVB/Blank: Photodamaged mice treated with MN;

UVB/CS/MN: Photodamaged mice treated with CS/MN;

UVB/ZnCS/MN: Photodamaged mice treated with ZnCS/MN.


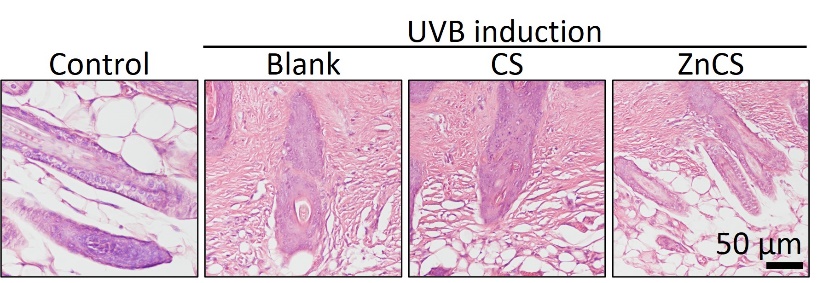


**Figure S5.** H&E staining of mouse skin at week 8 under high-magnification optical microscopy. Control: healthy mice; UVB/Blank: photodamaged mice treated with MN; UVB/CS/MN: photodamaged mice treated with CS/MN; UVB/ZnCS/MN: photodamaged mice treated with ZnCS/MN.

**
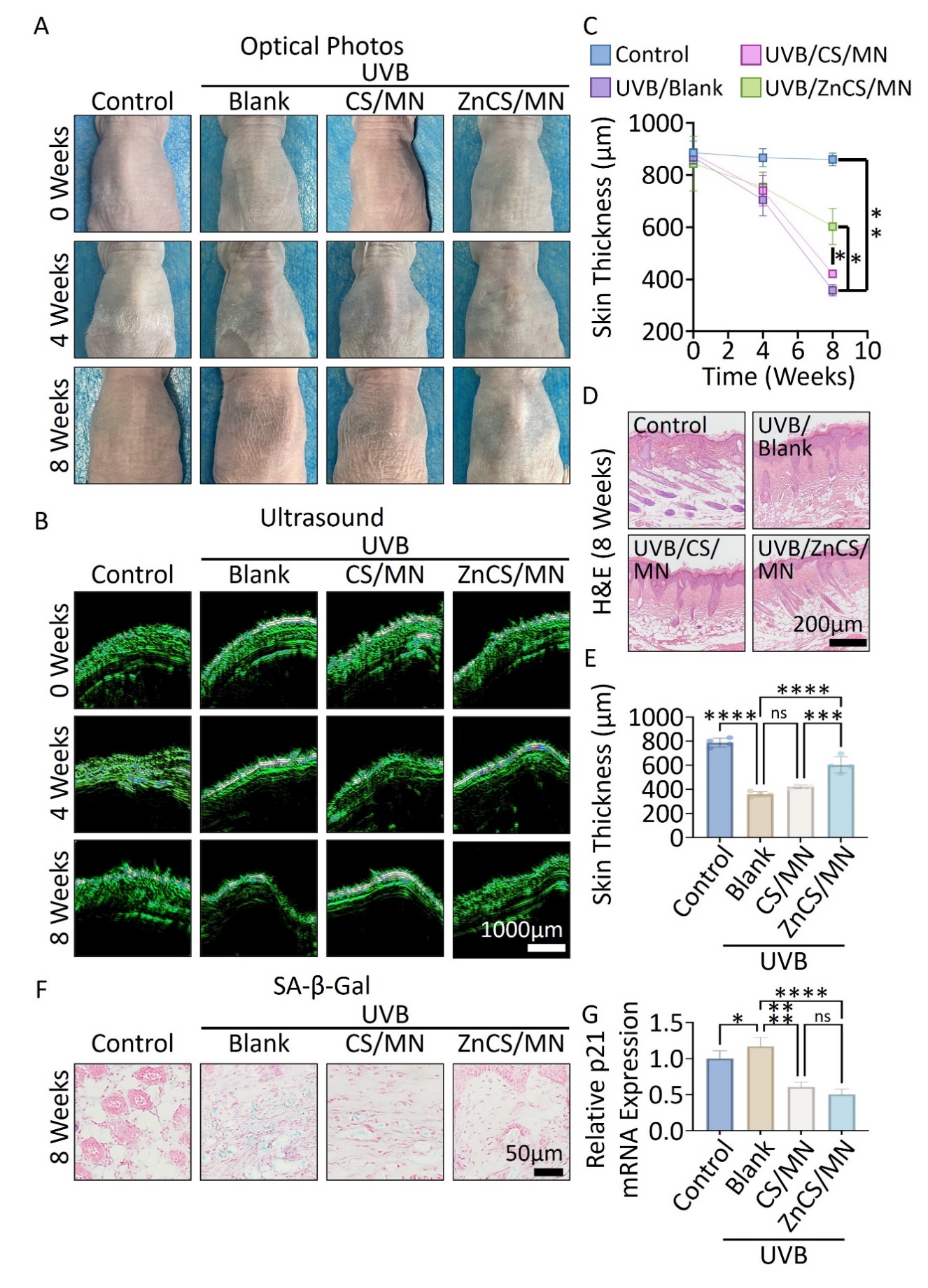
**

**Figure S6.** SA-β-Gal staining of mouse skin at week 8. Control: healthy mice; UVB/Blank: photodamaged mice treated with MN; UVB/CS/MN: photodamaged mice treated with CS/MN; UVB/ZnCS/MN: photodamaged mice treated with ZnCS/MN.


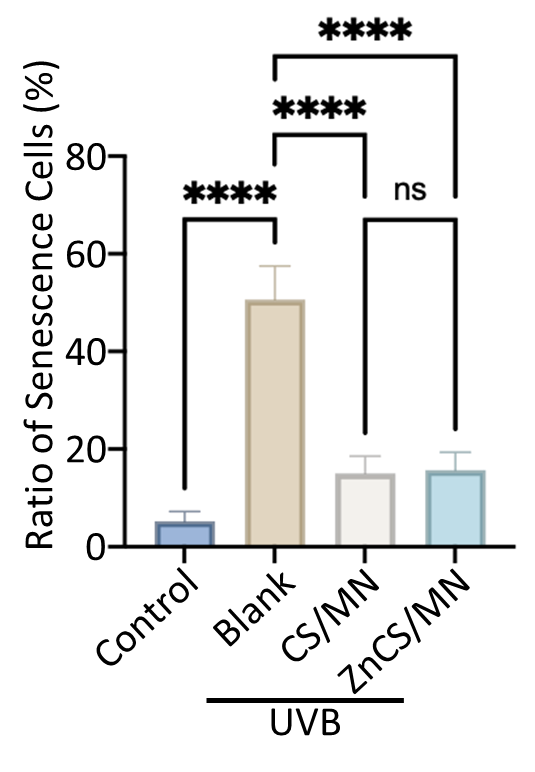


**Figure S7.** Quantitative analysis of the ratio of senescent cells according to SA-β-Gal staining. ****P < 0.0001, and ns: not significant. Control: healthy mice; UVB/Blank: photodamaged mice treated with MN; UVB/CS/MN: photodamaged mice treated with CS/MN; UVB/ZnCS/MN: photodamaged mice treated with ZnCS/MN.


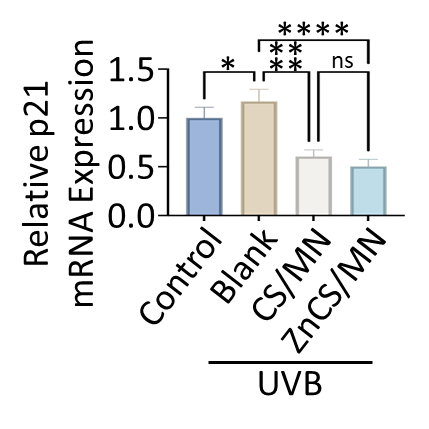


**Figure S8.** Relative mRNA expression of p21 in mouse skin detected by RT‒qPCR (n=5). *p < 0.05, *P < 0.05, **P < 0.01 ***P < 0.001, ****P < 0.0001, and ns: not significant. Control: healthy mice; UVB/Blank: photodamaged mice treated with MN; UVB/CS/MN: photodamaged mice treated with CS/MN; UVB/ZnCS/MN: photodamaged mice treated with ZnCS/MN.


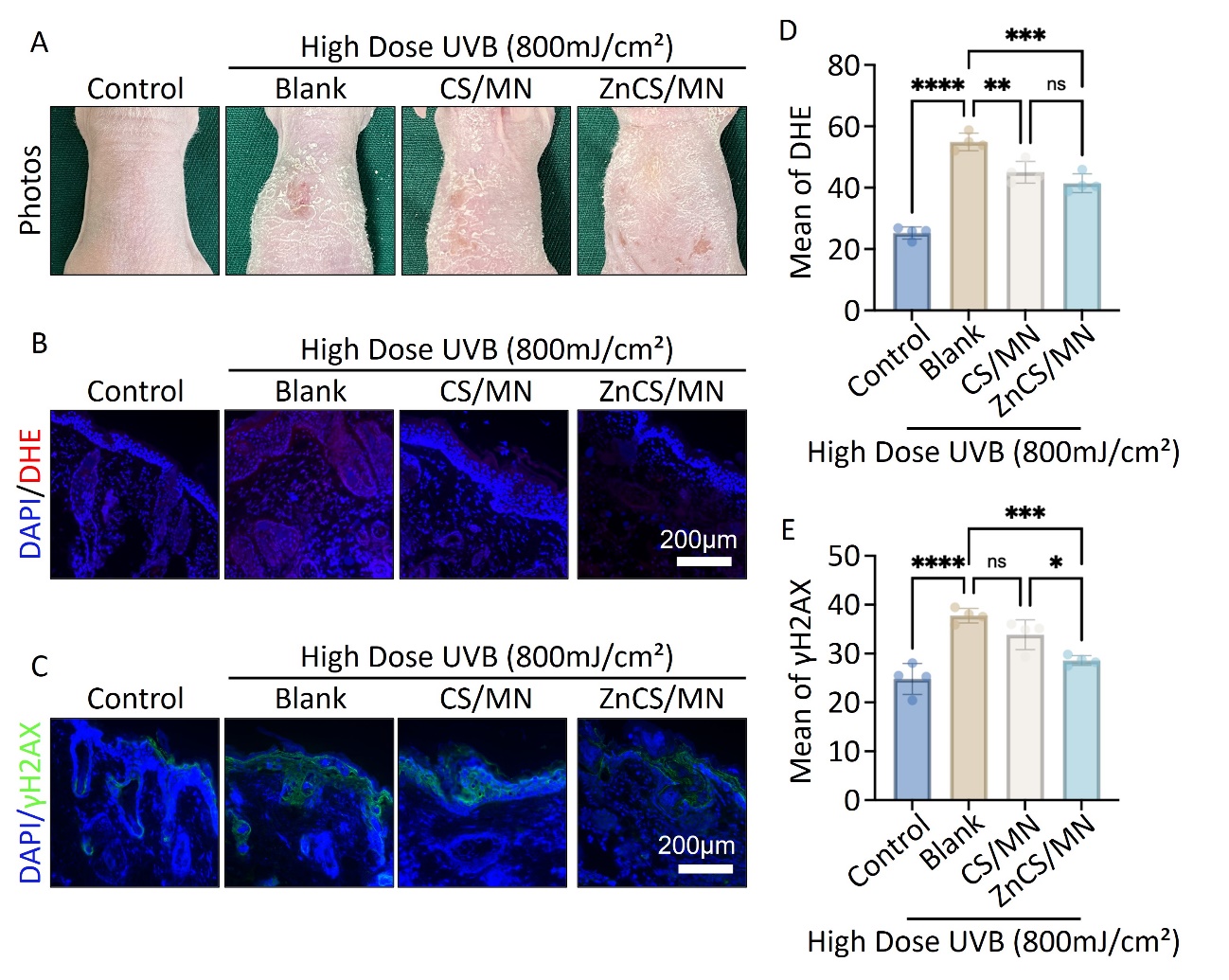


**Figure S9.** Effects of ZnCS/MN on acute photodamaged mice. (A) Skin appearance of mice subjected to different interventions. (B) Immunofluorescence staining of DHE (FITC: red; DAPI: blue). (C) Quantitative analysis of the relative fluorescence intensity of ROS (DHE) (n=4). (D) Immunofluorescence staining of γH2AX (γH2AX: green; DAPI: blue). (E) Quantitative analysis of the relative fluorescence intensity of γH2AX (n=4). *p < 0.05, *P < 0.05, **P < 0.01, ***P < 0.001, ****P < 0.0001, and ns: not significant. Control: healthy mice; UVB/Blank: photodamaged mice treated with MN; UVB/CS/MN: photodamaged mice treated with CS/MN; UVB/ZnCS/MN: photodamaged mice treated with ZnCS/MN.


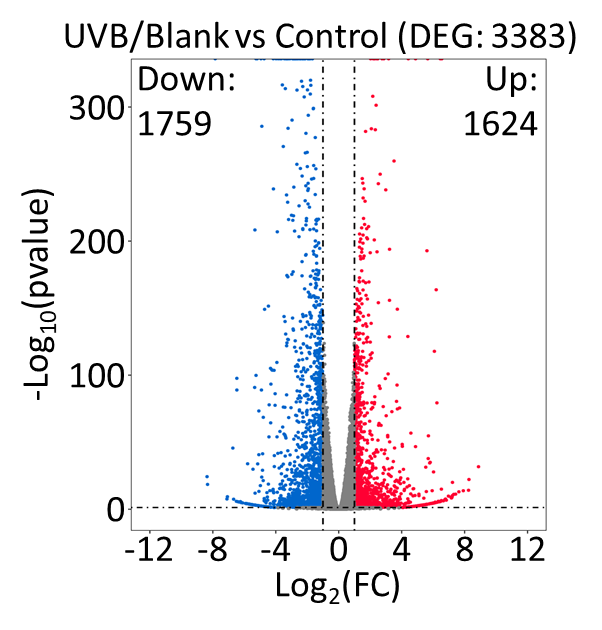


**Figure S10.** Volcano plots showing the number of DEGs in the UVB/blank and control groups. Control: Fibroblasts cultured in normal cell culture medium; UVB/Blank: Photodamaged fibroblasts cultured in normal cell culture medium.


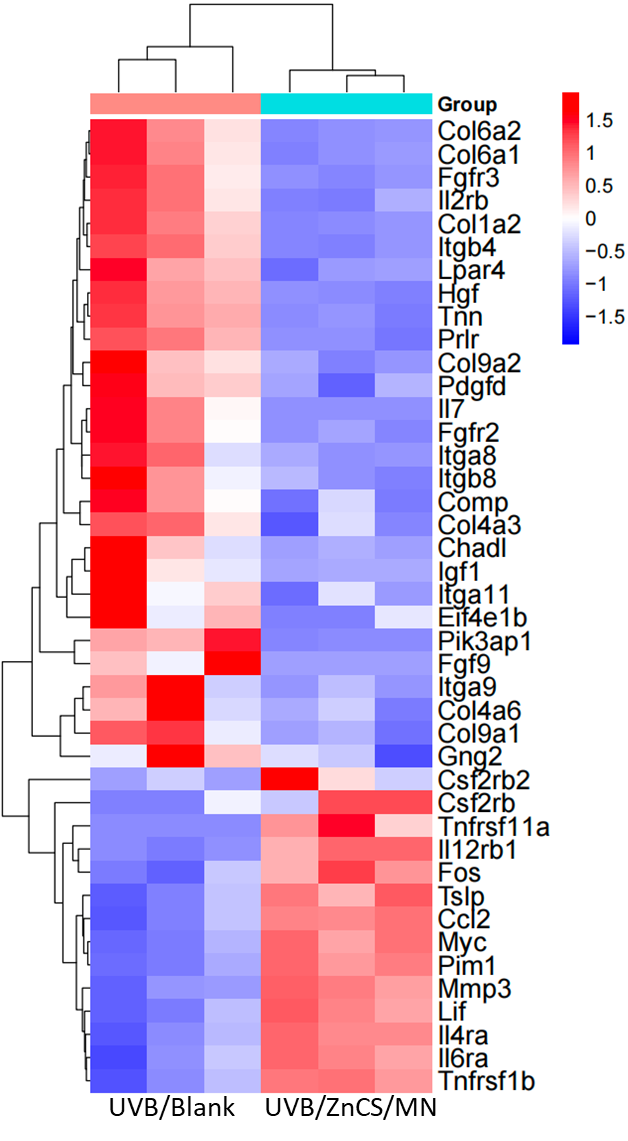

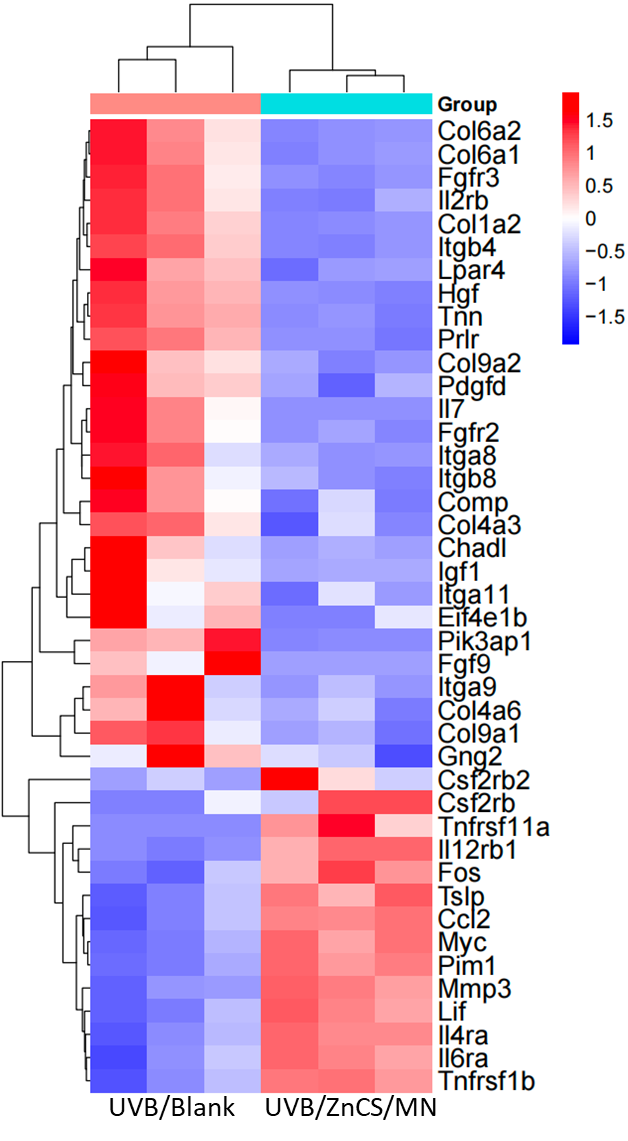

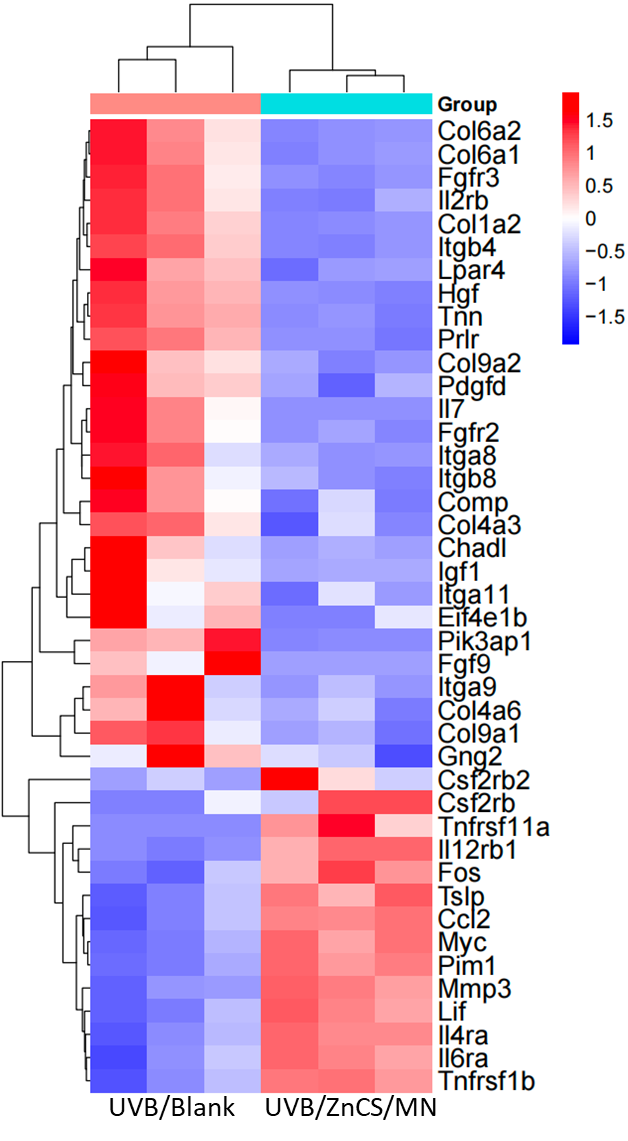


**Figure S11.** Heatmap showing the DEGs in the UVB/blank and UVB+ZnCS groups. UVB/Blank: Photodamaged mice treated with MN; UVB/ZnCS/MN: Photodamaged mice treated with ZnCS/MN.


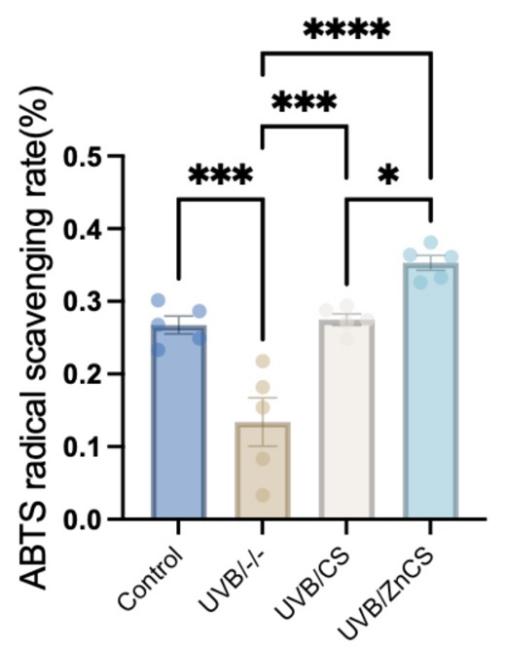


**Figure S12.** ABTS assay for total cellular antioxidant capacity. *p < 0.05, *P < 0.05, **P < 0.01, ***P < 0.001, ****P < 0.0001, and ns: not significant. Control: Fibroblasts cultured in normal cell culture medium; UVB/Blank: Photodamaged fibroblasts cultured in normal cell culture medium; UVB/CS: Photodamaged fibroblasts cultured in CS extract; UVB/ZnCS: Photodamaged fibroblasts cultured in ZnCS extract


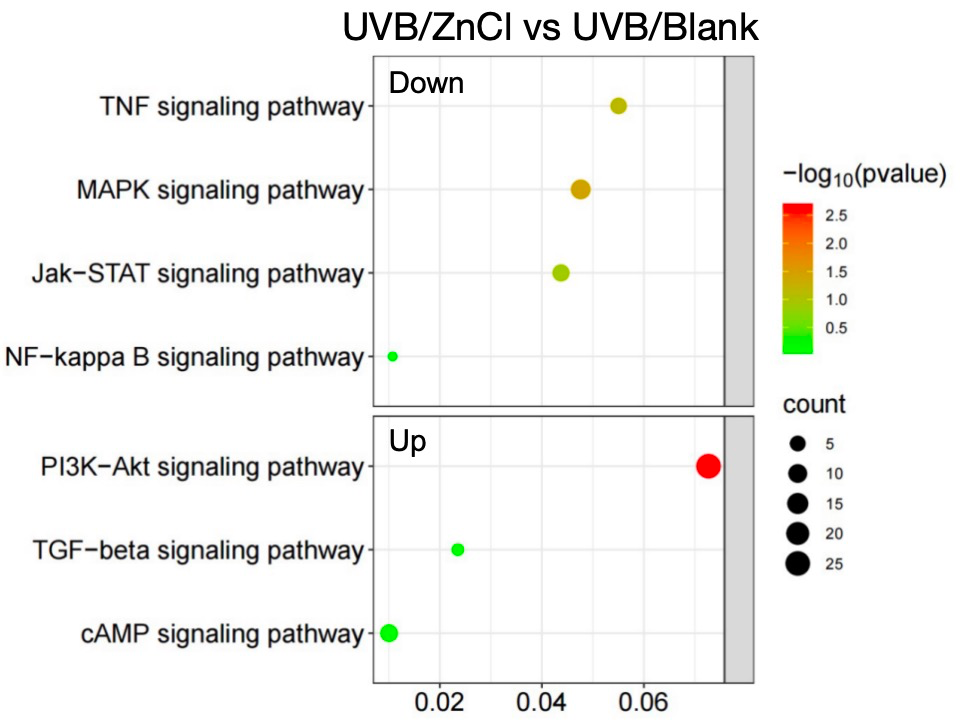


**Figure S13.** (A) Upregulated and downregulated pathways identified via KEGG analysis in the ZnCl/UVB group compared with the UVB/Blank group. UVB/Blank groups: Photodamaged fibroblasts cultured in normal cell culture medium; ZnCl/UVB groups: Photodamaged fibroblasts cultured in ZnCl/CS extract.


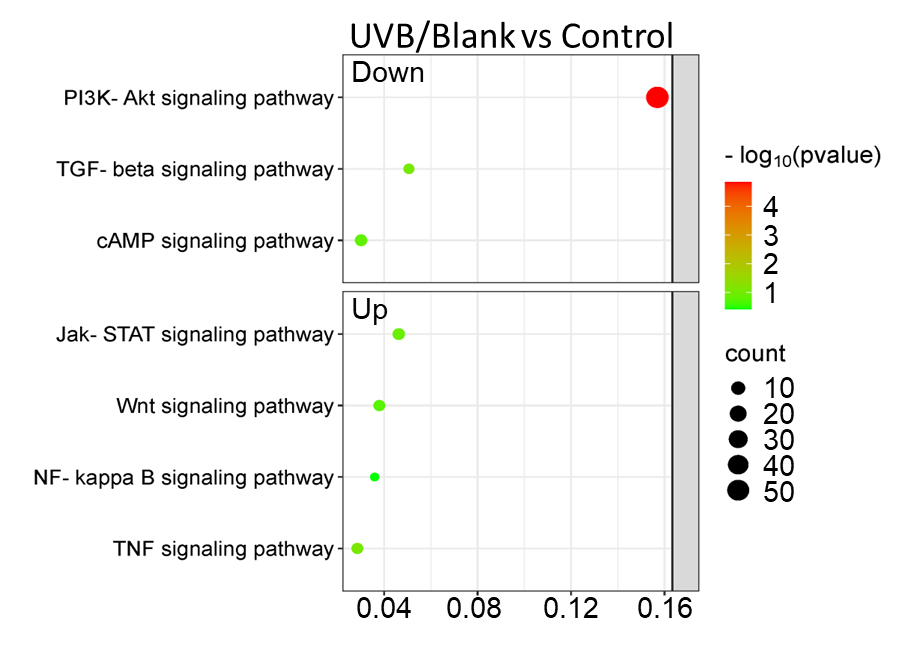


**Figure S14.** (A) Upregulated and downregulated pathways identified via KEGG analysis in the UVB/blank group compared with the control group. Control: healthy mice; UVB/Blank: photodamaged mice treated with MNs.


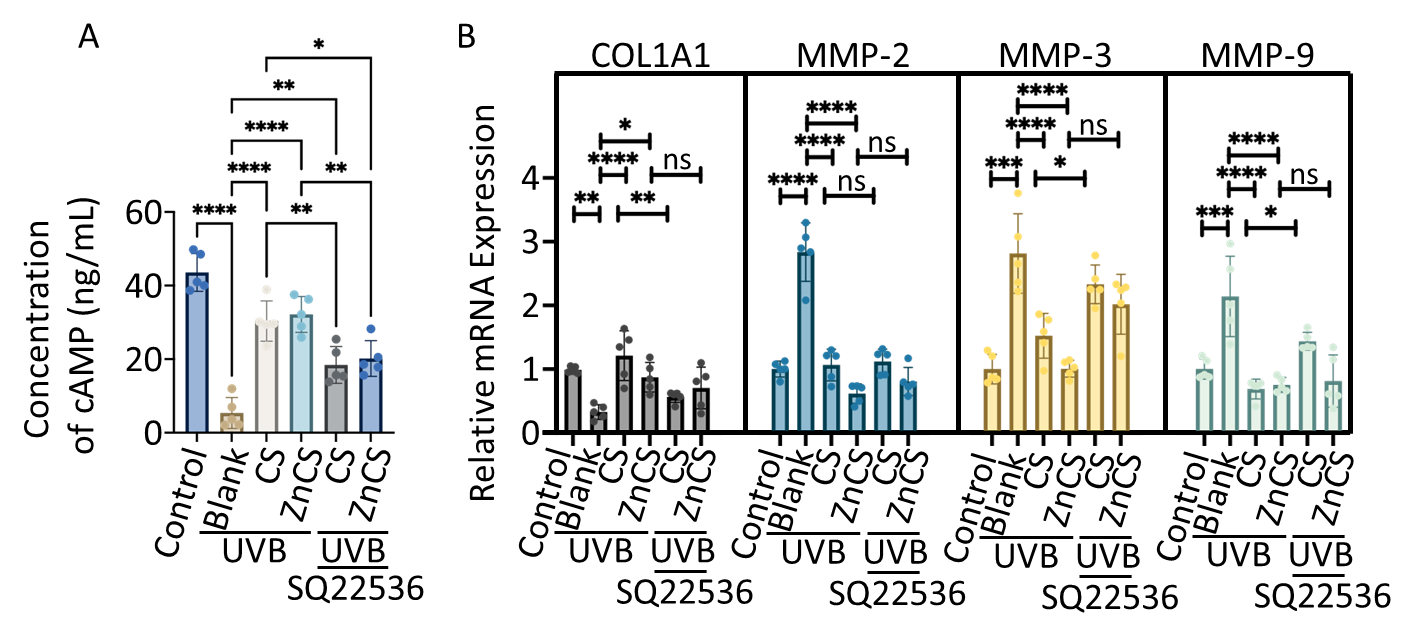


**Figure S15.** (A) Quantitative analysis of cAMP in cells via ELISA (n=5). (B) Quantitative analysis of the relative mRNA expression of COL1A1, elastin, MMP-2, MMP-3, and MMP-9 by RT‒qPCR (n=5). *p < 0.05, *P < 0.05, **P < 0.01, ***P < 0.001, ****P < 0.0001, and ns: not significant. Control: Fibroblasts cultured in normal cell culture medium; UVB/Blank: Photodamaged fibroblasts cultured in normal cell culture medium; UVB/CS: Photodamaged fibroblasts cultured in CS extract; UVB/ZnCS: Photodamaged fibroblasts cultured in ZnCS extract; UVB/CS/SQ22536: Photodamaged fibroblasts cultured in CS extract and SQ22536; UVB/ZnCS/SQ22536: Photodamaged fibroblasts cultured in ZnCS extract and SQ22536; CS extract dilution ratio: 1/32; ZnCS extract dilution ratio: 1/32.

**Table S1.**

| The Ion Concentration in CS Extract (μg/mL) | | |
| --- | --- | --- |
| Extract Ratio | Concentration of Zn^2+^ | Concentration of SiO_3_^2-^ |
| 1/4 | - | 33.11±1.71 |
| 1/8 | - | 16.29±0.63 |
| 1/16 | - | 7.96±0.11 |
| 1/32 | - | 4.06±0.27 |
| 1/64 | - | 2.03±0.12 |
| The Ion Concentration in ZnCS Extract (μg/mL) | | |
| Extract Ratio | Concentration of Zn^2+^ | Concentration of SiO_3_^2-^ |
| 1/4 | 7.73±0.41 | 39.55±2.25 |
| 1/8 | 3.92±0.17 | 19.57±0.56 |
| 1/16 | 1.96±0.08 | 9.75±0.35 |
| 1/32 | 0.96±0.06 | 5.13±0.05 |
| 1/64 | 0.49±0.03 | 2.33±0.07 |

**Table S2. Primers used in the experiment.**

| Gene | Forward 5‘-3’ | Reverse 5‘-3’ | Strain |
| --- | --- | --- | --- |
| GAPDH | AAGCTGTGGCGTGATGGC | TGACCTTGCCCACAGCCT | Mouse |
| COL1A1 | CCACAATGGCACGGCTGT | AAAGCACAGCACTCGCCC | Mouse |
| MMP-2 | ACCATGCGGAAGCCAAGATGTG | AGGGTCCAGGTCAGGTGTGTAAC | Mouse |
| MMP-3 | TTCTGGGCTATACGAGGGCA | CTTCTTCACGGTTGCAGGGA | Mouse |
| MMP-9 | GCCGACTTTTGTGGTCTTCC | GGTACAAGTATGCCTCTGCCA | Mouse |
| Elastin | TTGCTGATCCTCTTGCTCAAC | GCCCCTGGATAATAGACTCCAC | Mouse |
| IL-6 | ACAACCACGGCCTTCCC | AGCCTCCGACTTGTGAA | Mouse |
| IL-8 | TCCAATTCGGGAGACCTCTA | TAGGCATCACTGCCTGTCAA | Mouse |
| TNFα | CGCTCTTCTGTCTACTGAACTTCGG | GTGGTTTGTGAGTGTGAGGGTCTG | Mouse |

**Table S3.** **Gene Ontology (GO) Enrichment Analysis**

| Term | Count | Pvalue | group |
| --- | --- | --- | --- |
| response to external stimulus | 74 | 1.6×10⁻⁸ | UVB/CS/MN vs UVB/Blank |
| defense response | 54 | 2.5×10⁻⁸ |  |
| regulation of multicellular organismal process | 81 | 3.0×10⁻⁸ |  |
| cellular response to chemical stimulus | 80 | 7.7×10⁻⁸ |  |
| immune system process | 73 | 1.5×10⁻⁷ |  |
| response to biotic stimulus | 41 | 1.7×10⁻⁷ |  |
| response to cytokine | 35 | 1.9×10⁻⁷ |  |
| response to lipid | 45 | 2.8×10⁻⁷ |  |
| muscle filament sliding | 6 | 3.1×10⁻⁷ |  |
| lymph node development | 7 | 3.4×10⁻⁷ |  |
| monovalent inorganic cation transport | 13 | 1.3×10⁻⁵ | UVB/ZnCS/MN vs UVB/Blank |
| sodium ion transport | 8 | 9.3×10⁻⁵ |  |
| response to peptide | 13 | 1.1×10⁻⁴ |  |
| response to insulin | 9 | 1.7×10⁻⁴ |  |
| response to peptide hormone | 12 | 2.0×10⁻⁴ |  |
| muscle filament sliding | 3 | 2.2×10⁻⁴ |  |
| actin-myosin filament sliding | 3 | 2.5×10⁻⁴ |  |
| bile acid biosynthetic process | 3 | 3.3×10⁻⁴ |  |
| steroid biosynthetic process | 6 | 6.0×10⁻⁴ |  |
| response to vitamin B6 | 2 | 7.2×10⁻⁴ |  |
| hard palate development | 2 | 7.2×10⁻⁴ | UVB/ZnCS/MN vs UVB/Blank |
| chromosome segregation | 44 | 2.5×10⁻²⁴ |  |
| cell division | 52 | 1.3×10⁻²⁰ |  |
| nuclear chromosome segregation | 35 | 4.3×10⁻¹⁹ |  |
| nuclear division | 39 | 1.8×10⁻¹⁶ |  |
| cell cycle process | 66 | 3.9×10⁻¹⁶ |  |
| mitotic nuclear division | 30 | 6.0×10⁻¹⁶ |  |
| mitotic sister chromatid segregation | 23 | 1.5×10⁻¹⁵ |  |
| mitotic cell cycle process | 48 | 2.5×10⁻¹⁵ |  |
| sister chromatid segregation | 26 | 2.9×10⁻¹⁵ |  |
| organelle fission | 39 | 4.9×10⁻¹⁵ |  |
| mitotic cell cycle | 50 | 3.3×10⁻¹⁴ |  |
